# 4‐Hydroxybenzyl Alcohol Mitigates Hyperlipidemia‐Associated Depression by Inhibiting Neuroinflammation via the NKIRAS2/NF‐κB Pathway

**DOI:** 10.1002/advs.202517873

**Published:** 2026-03-09

**Authors:** Ying Zhang, Meng Teng, Wenxuan He, Lanzhou Li, Yongfeng Zhang, Shimiao Wang, Chunyue Wang, Di Wang

**Affiliations:** ^1^ School of Life Sciences Jilin University Changchun China; ^2^ Engineering Research Center of Chinese Ministry of Education for Edible and Medicinal Fungi School of Plant Protection Jilin Agricultural University Changchun China

**Keywords:** 4‐hydroxybenzyl alcohol, depression, hyperlipidemia, neuroinflammation, NKIRAS2/NF‐κB pathway

## Abstract

Epidemiological data link hyperlipidemia to increased depression susceptibility. This study investigates the potential involvement of 4‐hydroxybenzyl alcohol (4‐HBA), a bioactive molecule known for its neuroprotective and anti‐inflammatory effects, in the pathophysiology of hyperlipidemia‐associated depression. High‐fat diet (HFD)‐fed mice develop concurrent hyperlipidemia and depression‐like behaviors, with 4‐HBA identified as a key modulated brain metabolite in fecal microbiota transplantation recipients. In HFD‐fed mice, 4‐HBA treatment simultaneously improves lipid metabolism and significantly alleviates depression‐like behaviors, accompanied by suppression of the nuclear factor κB (NF‐κB) signaling pathway in the brain. In LPS‐stimulated BV2 cells, 4‐HBA inhibits NF‐κB activation through NF‐κB inhibitor interacting Ras‐like 2 (NKIRAS2), thereby coordinating the downregulation of inflammatory responses. Conditioned medium from 4‐HBA‐treated BV2 cells enhances neuronal viability and reduces inflammatory responses in HT22 neurons in co‐culture. Importantly, silencing *Nkiras2* in BV2 cells and organotypic brain slice cultures negated the anti‐inflammatory and neuroprotective actions of 4‐HBA. These findings demonstrate that the NKIRAS2/NF‐κB pathway is a molecular mediator underlying the biological effects of 4‐HBA. These findings position 4‐HBA as a dual‐action metabolite capable of concurrently mitigating metabolic and psychiatric manifestations through neuroinflammatory regulation.

## Graphical abstract

4‐HBA upregulates NKIRAS2 levels, inhibiting the activation of the NF‐κB pathway and subsequently reducing the levels of neuroinflammatory markers. This modulation helps restore normal mood and behavior in hyperlipidemic conditions, providing a potential therapeutic strategy for managing hyperlipidemia‐associated depression.

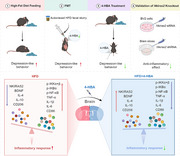

## 1 Introduction

High‐fat diet (HFD) contributes to the pathogenesis of various human diseases, including hyperlipidemia [1]. A 2000–2025 meta‐analysis found global prevalences of 28.8% for hypertriglyceridemia, 24.1% for hypercholesterolemia, 38.4% for low high‐density lipoprotein cholesterol (HDL‐C), and 18.93% for high low‐density lipoprotein cholesterol (LDL‐C) [2]. Individuals with hyperlipidemia exhibit a markedly higher risk of developing depression [3]. Large‐scale cohort studies consistently link high triglyceride (TG) levels with increased long‐term risk of depression, anxiety, and stress‐related disorders [4]. This suggests that lipid dysregulation may play a pivotal role in the onset of common mental health conditions. Patients with depression exhibit adverse lipid profiles associated with metabolic disturbances [5]. However, current clinical practice fails to address this comorbidity systematically, highlighting a critical gap requiring unified treatment protocols.

Although these epidemiological associations are well‐established, the underlying pathological mechanisms linking hyperlipidemia and depression remain elusive. Current evidence supports an interplay among chronic low‐grade inflammation, depressive symptoms, and dyslipidemia, with pro‐inflammatory cytokines contributing to depressive symptoms, promoting metabolic disturbances, and remaining elevated in both depressed and obese individuals [6, 7]. In depression, hyperactivation of the hypothalamic‐pituitary‐adrenal (HPA) axis promotes excessive corticosterone secretion, thereby contributing to hyperlipidemia [8]. Concomitant inflammatory responses further potentiate HPA axis activity, forming a vicious cycle that exacerbates lipid dysregulation [9]. Furthermore, dysregulation of intracellular signaling pathways has been identified as a crucial mediator linking metabolic disorders to depression‐like behaviors. For example, prolonged consumption of a saturated fat‐rich HFD induces systemic metabolic inflammation by activating nuclear factor κB (NF‐κB) signaling in the nucleus accumbens (NAc), thereby promoting neuroinflammation. Notably, targeted blockade of the NF‐κB pathway in the NAc alleviates HFD‐induced anxiety‐depressive behaviors in rodents, highlighting its pivotal role in linking unhealthy dietary patterns and metabolic dysregulation to the development of affective disorders [10]. The pathophysiology of depression associated with metabolic disorders cannot be attributed solely to pro‑inflammatory signaling. Alongside NF‐κB activation, anti‑inflammatory and homeostatic pathways play an essential role in regulating neuroimmune balance. Among these, the transforming growth factor β/SMAD (TGF‐β/SMAD) signaling pathway plays a key role in energy homeostasis and insulin sensitivity and is also implicated in depression pathophysiology [11]. This connection is mediated through its influence on neuroplasticity, stress adaptation, and the functioning of emotion‐related brain regions such as the amygdala [12, 13]. These findings define a framework in which neuroinflammatory, neuroendocrine, and neurometabolic disturbances jointly contribute to the comorbidity of hyperlipidemia and depression.

Metabolites derived from gut microbiota are recognized as key exogenous regulators of the inflammatory and stress‐related signaling pathways. Sodium butyrate has been shown to exert neuroprotective effects by suppressing NF‐κB signaling activity and enhancing both peripheral and central metabolic homeostasis [14]. Similarly, spermidine ameliorated HFD‐induced obesity by enhancing lipolysis in visceral adipose tissue [15]. It also normalized microglial neuroinflammation through regulation of toll‐like receptor (TLR)3/4 signaling [16]. Among these promising metabolites, 4‐hydroxybenzyl alcohol (4‐HBA) stood out due to its capacity to coordinately regulate neuroinflammation and oxidative stress. 4‐HBA demonstrated multifaceted neuroprotective effects by simultaneously suppressing lipopolysaccharide (LPS)‐induced proinflammatory cytokine release [17], restoring neurotrophic factor expression to prevent neuronal and behavioral impairments [18], and reducing oxidative damage through antioxidant pathway activation in brain injury [19]. The documented biological activities of 4‐HBA are largely confined to neuroprotective effects. Nevertheless, its involvement in metabolic mood disorders still awaits systematic investigation.

In the present study, we employed a multilevel experimental framework encompassing HFD‐fed mice, fecal microbiota transplantation (FMT) models, LPS‐stimulated BV2 cells, BV2‐HT22 conditioned medium co‐culture, and organotypic brain slice cultures (OBSCs) to demonstrate that 4‐HBA ameliorated hyperlipidemia‐associated depressive phenotypes by integrating behavioral assessments with biochemical analyses. The NF‐κB inhibitor interacting Ras‐like protein 2 (NKIRAS2)/NF‐κB signaling pathway was identified as a critical mediator of the anti‐inflammatory effects of 4‐HBA. A bioinformatic analysis of human transcriptomic datasets was conducted to identify conserved pathogenic pathways and validate the clinical relevance of the experimental observations. These findings demonstrated that 4‐HBA exerted ameliorative effects against hyperlipidemia‐depression comorbidity and supported its therapeutic potential for this intersecting metabolic and psychiatric condition.

## 2 Results

### 2.1 HFD‐Fed Mice Exhibited Depression‐Like Behaviors and Brain Damage

**FIGURE 1:**
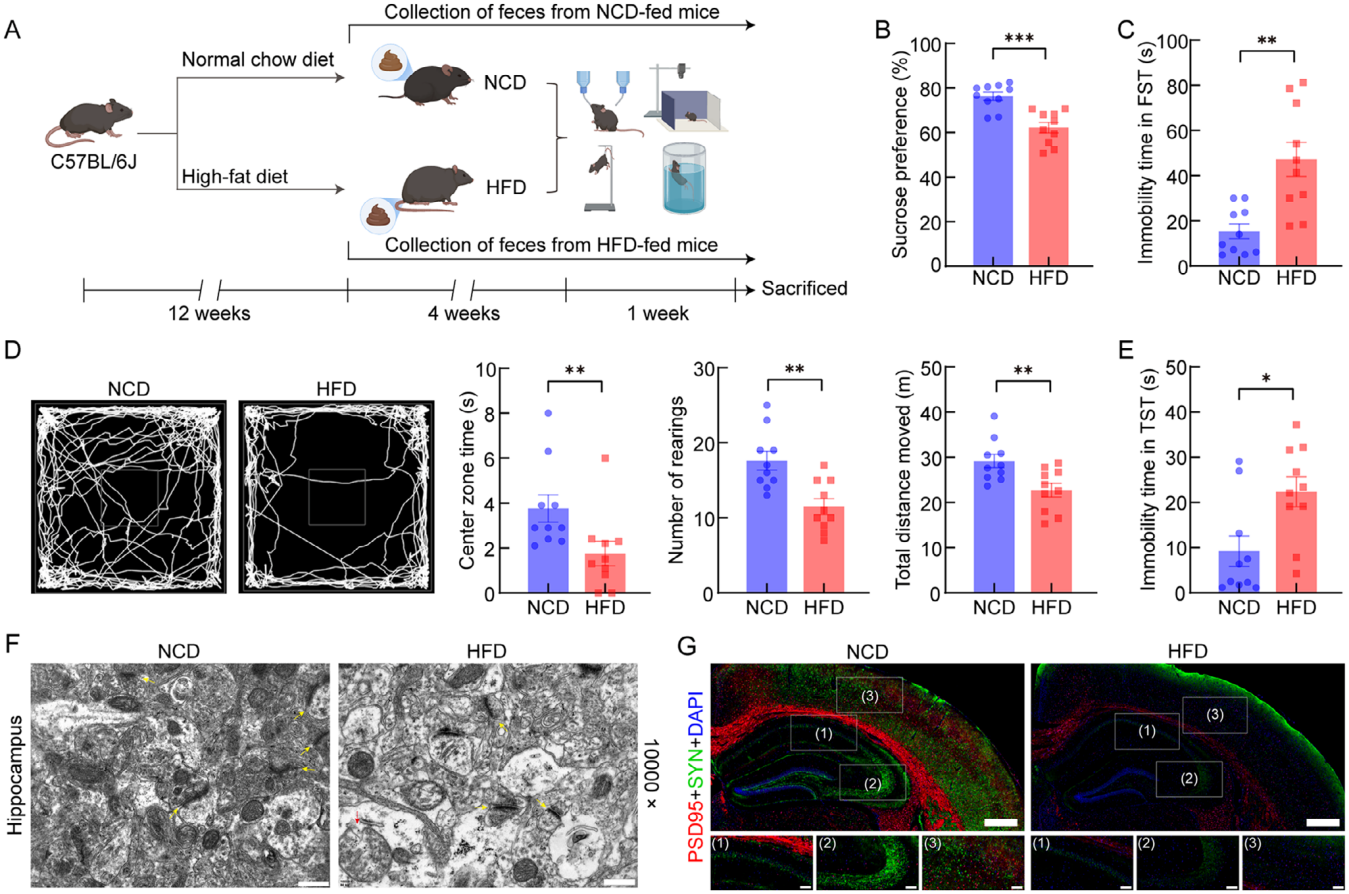
HFD‐fed mice exhibited depression‐like behaviors and brain damage. (A) Schematic of the HFD mouse model experimental design. HFD feeding significantly (B) reduced sucrose preference (*p* = 0.0002; *n* = 10), (C) increased immobility time in the FST (*p* = 0.0021; *n* = 10), (D) decreased center zone time (*p* = 0.0050; *n* = 10), number of rearings (*p* = 0.0015; *n* = 10), and total distance moved (*p* = 0.0073; *n* = 10) in the OFT, and (E) prolonged immobility time in the TST (*p* = 0.0156; *n* = 10). (F) TEM of hippocampus (10 000×, scale bar: 500 nm; *n* = 3). Yellow arrows indicate synaptic structures; red arrows indicate damaged mitochondria. (G) Representative images showed PSD95 and SYN expression in CA1 and CA3 regions and the cortex, with PSD95‐positive cells labeled in red and SYN‐positive cells labeled in green (40×, scale bar: 500 µm; 200×, scale bar: 100 µm; *n* = 3). Intergroup comparisons were analyzed using a two‐tailed unpaired Student's *t*‐test or Mann–Whitney *U* test to determine statistical significance. Data are expressed as mean ± standard error of the mean (SEM). ^*^ *p* < 0.05, ^**^ *p* < 0.01, and ^***^ *p* < 0.001 versus NCD‐fed mice.

Mice were assigned to a normal chow diet (NCD) and HFD groups and subjected to dietary intervention (Figure 1A). Relative to NCD‐fed mice, HFD‐fed mice displayed a pronounced dyslipidemic phenotype, characterized by significant increases in serum total cholesterol (TC), TG, and LDL‐C, accompanied by a marked reduction in HDL‐C (Figure S1A–D). These findings demonstrated that chronic high‐fat feeding induced profound dysregulation of systemic lipid metabolism, which verified the successful establishment of the hyperlipidemic mouse model. Given the well‐established evidence that metabolic disorders increase the risk of depression and anxiety [20], we further investigated whether these metabolic disorders were accompanied by alterations in emotional behavior. Through a series of validated behavioral tests, we observed that chronic HFD consumption for 16 weeks significantly promoted depression‐like behaviors in mice. Specifically, HFD‐fed mice demonstrated markedly reduced sucrose preference relative to NCD‐fed mice, indicating anhedonia‐like behavior (Figure 1B). HFD‐fed mice exhibited significantly increased immobility time in the forced swim test (FST) compared to the NCD‐fed mice (Figure 1C). In the open field test (OFT), HFD feeding significantly decreased the time spent in the center zone, number of rearings, and total distance moved (Figure 1D). HFD‐fed mice exhibited increased immobility time in the tail suspension test (TST) compared with NCD‐fed mice (Figure 1E). Comprehensive behavioral tests consistently demonstrated significant depression‐like behaviors in HFD‐fed mice.

Transmission electron microscopy (TEM) analysis revealed that HFD‐fed mice exhibited significant synaptic loss compared to NCD‐fed mice, along with ultrastructural aberrations in mitochondria, including disrupted integrity of both inner and outer mitochondrial membranes and fragmentation of cristae (Figure 1F). These ultrastructural pathologies provided direct visual evidence of HFD‐induced neuronal damage at nanoscale resolution. The presynaptic marker synaptophysin (SYN) and postsynaptic density protein 95 (PSD95) serve as key molecular biomarkers for monitoring synaptic development and stability [21]. Immunofluorescence (IF) analysis of brain tissues revealed significantly reduced expression of SYN and PSD95 in HFD‐fed mice (Figure 1G; Figure S2A–F). The parallel decline of these synaptic markers corroborated the synaptic degeneration observed by TEM.

### 2.2 HFD‐FMT Induced Depression‐Like Behaviors With Brain Metabolome Alterations

**FIGURE 2:**
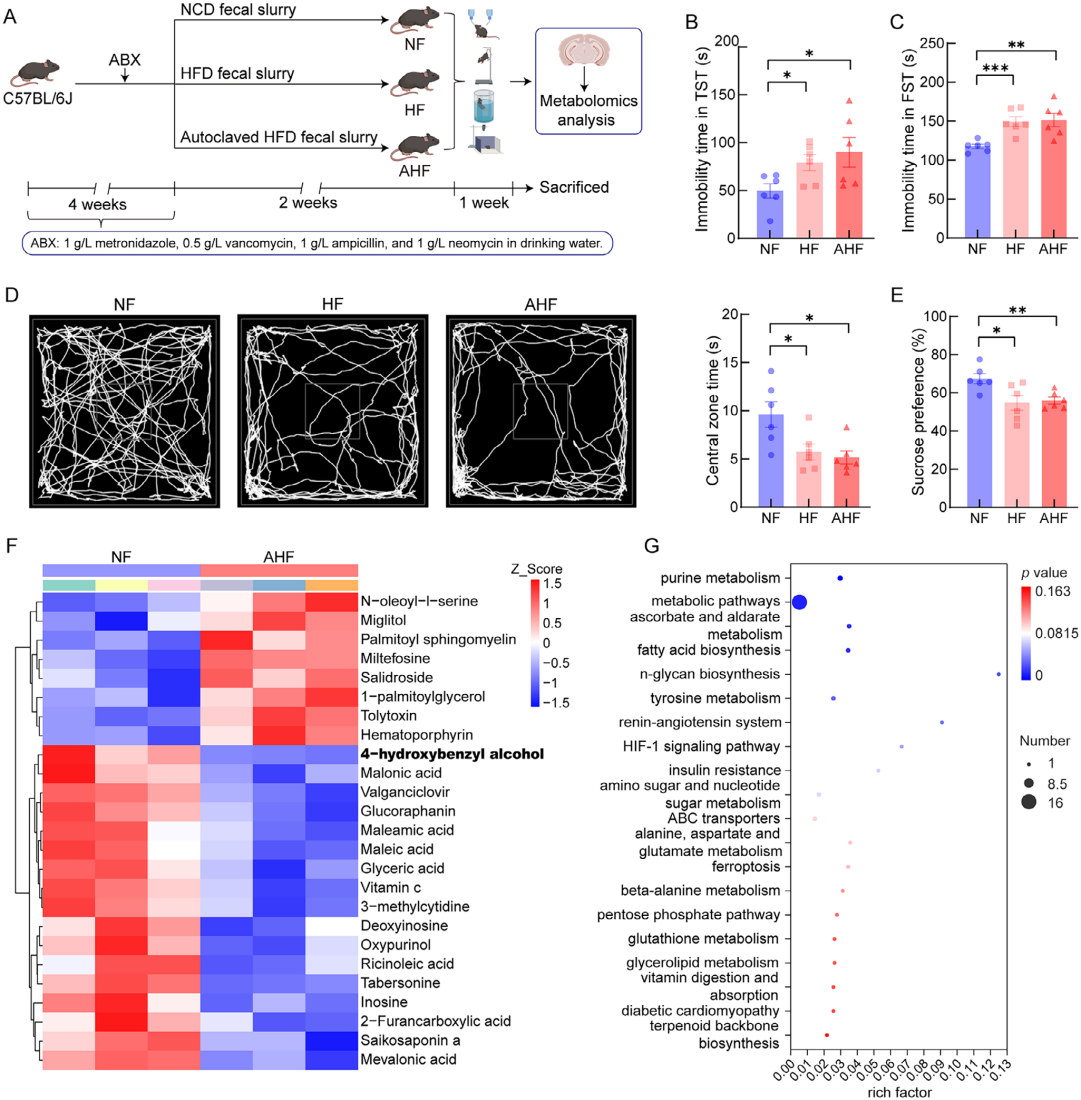
HFD‐FMT induced depression‐like behaviors with brain metabolome alterations. (A) Schematic of the FMT experimental design in mice. Compared with NF mice, both HF and AHF mice exhibited a significant increase in (B) immobility time in the TST (HF vs. NF: *p* = 0.0244; AHF vs. NF: *p* = 0.0492; *n* = 6) and (C) FST (HF vs. NF: *p* = 0.0009; AHF vs. NF: *p* = 0.0092; *n* = 6), (D) a reduction in center zone time in the OFT (HF vs. NF: *p* = 0.0321; AHF vs. NF: *p* = 0.0133; *n* = 6), and (E) a decrease in sucrose preference in the SPT (HF vs. NF: *p* = 0.0222; AHF vs. NF: *p* = 0.0047; *n* = 6). (F) Cluster heat map analysis of significantly differential metabolites between NF and AHF mice. (G) Bubble plot of KEGG pathway enrichment analysis between NF and AHF mice. Intergroup comparisons were analyzed using a two‐tailed unpaired Student's *t*‐test or Mann–Whitney *U* test to determine statistical significance. Data are expressed as mean ± SEM. ^*^ *p* < 0.05, ^**^ *p < *0.01, and ^***^ *p* < 0.001 versus NF mice.

Mice were divided into three groups receiving NCD fecal slurry (NF), HFD fecal slurry (HF), or autoclaved HFD fecal slurry (AHF) (Figure 2A). Serum lipid analysis showed no significant differences in TC, TG, LDL‐C, or HDL‐C among these groups (Figure S3A–D). Behavioral analyses demonstrated that, compared with NF mice, both HF and AHF mice exhibited significantly increased immobility time in the TST and FST (Figure 2B,C). HF and AHF mice also showed anxiety‐like phenotypes, as evidenced by significantly reduced center zone time in the OFT (Figure 2D). Furthermore, the sucrose preference test (SPT) revealed significantly diminished sucrose preference in both HF and AHF mice relative to NF mice (Figure 2E). These behavioral deficits collectively demonstrated that FMT using HFD fecal slurry and autoclaved HFD fecal slurry exacerbated depression‐like behaviors across multiple neurobehavioral domains. Given the significant behavioral differences between NF and AHF mice, we conducted untargeted metabolomics analysis of brain tissues. Metabolomic clustering analysis revealed significantly reduced 4‐HBA levels in AHF group brains compared to NF mice (Figure 2F; Table S1). Kyoto Encyclopedia of Genes and Genomes (KEGG) enrichment analysis identified significant enrichment of differential metabolites in several key pathways, including tyrosine metabolism, renin‐angiotensin system, and fatty acid biosynthesis (Figure 2G). Given the convergence of these pathways on inflammation, metabolism, and affect regulation, we next aimed to identify metabolites potentially causally linked to the depressive‐like phenotype in AHF mice. Among the metabolites significantly reduced following AHF treatment, 4‐HBA emerged as a strong mechanistic candidate. Previous in vivo investigations have demonstrated that 4‐HBA confers both anti‐inflammatory and neuroprotective effects [22, 23]. The finding of a pronounced reduction in brain 4‐HBA levels in AHF mice raised the possibility of its involvement in the depressive‐like phenotype. On this basis, we designated 4‐HBA as the primary target for subsequent mechanistic investigation.

### 2.3 4‐HBA Ameliorated Hyperlipidemia and Depressive‐Like Symptoms in HFD‐Fed Mice

**FIGURE 3:**
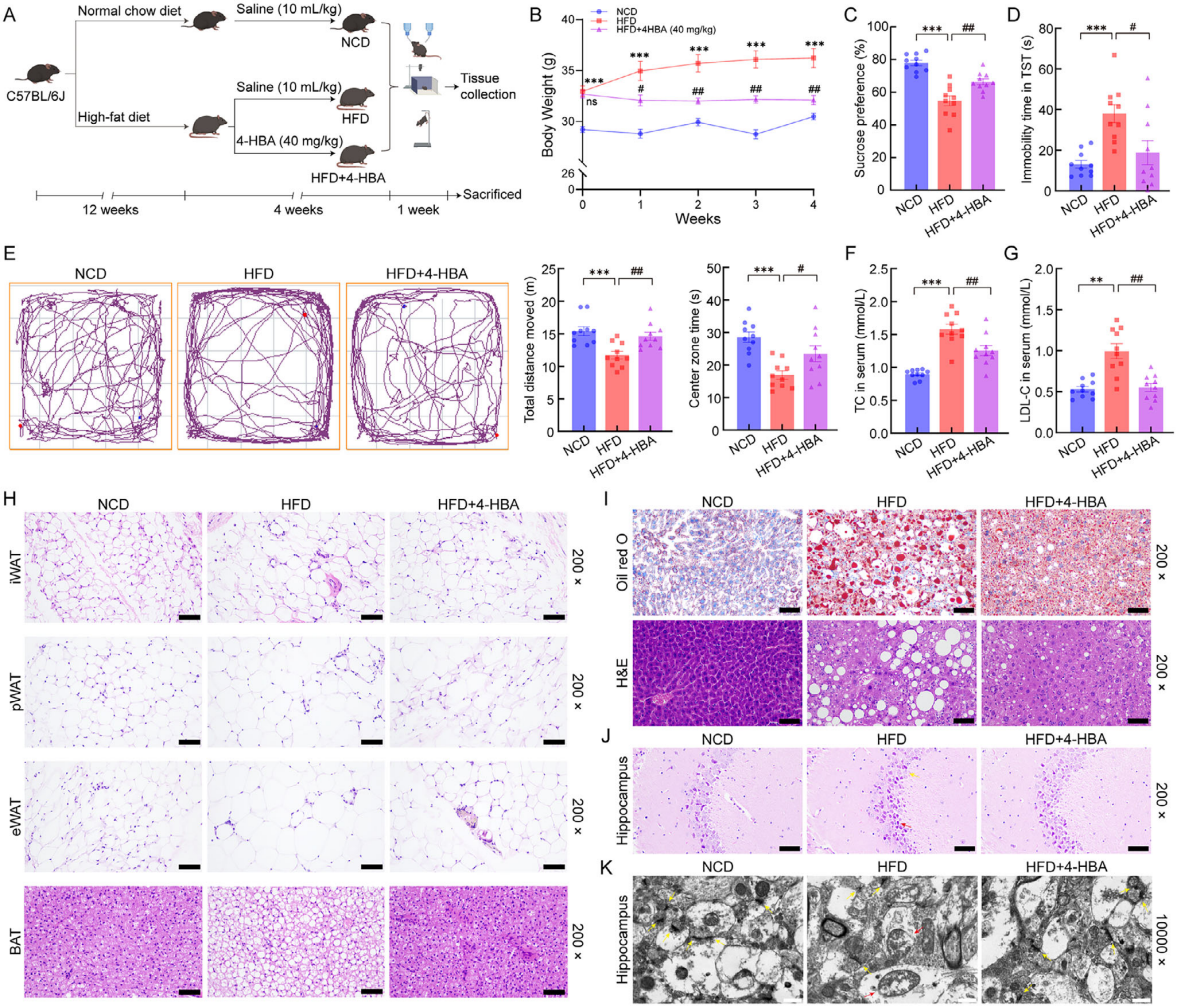
4‐HBA ameliorated depression‐like behaviors and brain damage in HFD‐fed mice. (A) Schematic of the 4‐HBA treatment experimental design. (B) 4‐HBA treatment significantly attenuated body weight gain in HFD‐fed mice (*p* = 0.0047; *n* = 10). (C) 4‐HBA restored sucrose preference in the SPT (*p* = 0.0033; *n* = 10). (D) 4‐HBA reduced immobility time in the TST (*p* = 0.0457; *n* = 10). (E) 4‐HBA increased total distance moved (*p* = 0.0087; *n* = 10) and center zone time in the OFT (*p* = 0.0484; *n* = 10). 4‐HBA significantly decreased serum (F) TC (*p* = 0.0034; *n* = 10) and (G) LDL‐C (*p* = 0.0021; *n* = 10) in HFD‐fed mice. (H) Hematoxylin and eosin (H&E) staining of four adipose tissues (200×, scale bar: 100 µm; *n* = 3). (I) Oil Red O and H&E staining of liver sections (200×, scale bar: 100 µm; *n* = 3). (J) H&E staining of the hippocampus (200×, scale bar: 100 µm; *n* = 3). Yellow arrows indicate intact neurons; red arrows indicate degenerating neurons. (K) TEM of hippocampus (10 000×, scale bar: 500 nm; *n* = 3). Yellow arrows indicate synaptic structures; red arrows indicate damaged mitochondria. Intergroup differences were analyzed using one‐way ANOVA with Tukey's post hoc test or Tamhane's T2 test. Data are expressed as mean ± SEM. ns: no statistically significant difference. ^**^ *p* < 0.01 and ^***^ *p* < 0.001 versus NCD‐fed mice; ^#^ *p* < 0.05 and ^##^ *p* < 0.01 versus HFD‐fed mice.

HFD‐fed mice were administered 4‐HBA for four weeks (Figure 3A), resulting in a significant reduction in body weight gain compared with vehicle‐treated HFD‐fed mice (Figure 3B). In behavioral tests, 4‐HBA administration normalized sucrose preference in the SPT (Figure 3C), reduced immobility time in the TST (Figure 3D), and increased both total distance moved and center zone time in the OFT (Figure 3E). Although 4‐HBA suppressed body weight gain relative to vehicle‐treated HFD‐fed mice, analysis of covariance (ANCOVA) controlling for body weight demonstrated that the behavioral improvements remained statistically significant after adjustment (Figure S4A–D). These findings indicated that the antidepressant‐like effects of 4‐HBA were not solely attributable to weight changes. Collectively, the results supported a potent effect of 4‐HBA in ameliorating behavioral impairments in HFD‐fed mice. Compared with NCD‐fed mice, HFD‐fed mice showed significantly elevated serum TC and LDL‐C levels, while 4‐HBA treatment attenuated these increases (Figure 3F,G). HFD feeding induced pronounced adipocyte hypertrophy, along with significant increases in adipose tissue index in inguinal white adipose tissue (iWAT), perirenal white adipose tissue (pWAT), and epididymal white adipose tissue (eWAT), while concomitantly reducing brown adipose tissue (BAT) index. Notably, treatment with 4‐HBA significantly attenuated these pathological alterations, indicating its potential to counteract HFD‐induced adipose tissue remodeling (Figure 3H; Figure S5A–D). Compared to NCD‐fed mice with preserved hepatocyte architecture, HFD‐fed mice developed severe steatosis marked by cytoplasmic vacuolization and excessive lipid droplet accumulation, pathological alterations that were significantly attenuated with 4‐HBA treatment (Figure 3I). In addition, HFD feeding significantly increased the liver index, and this increase was also substantially reduced by 4‐HBA administration treatment (Figure S5E). Quantitative analysis demonstrated that HFD feeding significantly increased the spleen index, which was effectively reversed by 4‐HBA treatment (Figure S5F). Neither HFD feeding nor 4‐HBA administration significantly altered the heart or kidney index (Figure S5G,H). Histological examination showed that the structural integrity of main organs like the heart, spleen, and kidney was maintained among all experimental groups (Figure S5I). Moreover, prolonged intraperitoneal administration of 4‐HBA for 6 months did not induce detectable pathological alterations in the brain, heart, liver, spleen, or kidneys (Figure S6), further supporting the favorable biosafety profile of 4‐HBA.

Significant histological abnormalities were observed in HFD‐fed mice, including disorganized brain neuronal alignment, widened intercellular spaces, and numerous shrunken, hyperchromatic neurons with irregular morphology. Notably, 4‐HBA treatment reduced these abnormalities, restoring near‐normal cytoarchitecture (Figure 3J). TEM analysis showed that 4‐HBA restored synaptic loss and ameliorated mitochondrial pathologies, including membrane integrity and cristae morphology in HFD‐fed mice (Figure 3K). Collectively, 4‐HBA alleviated both the behavioral and neuropathological effects of HFD‐induced metabolic dysfunction in mice, suggesting its potential relevance to metabolic‐associated neuropsychiatric disorders.

### 2.4 4‐HBA Attenuated Inflammation in HFD‐fed Mice and Cellular Models

**FIGURE 4:**
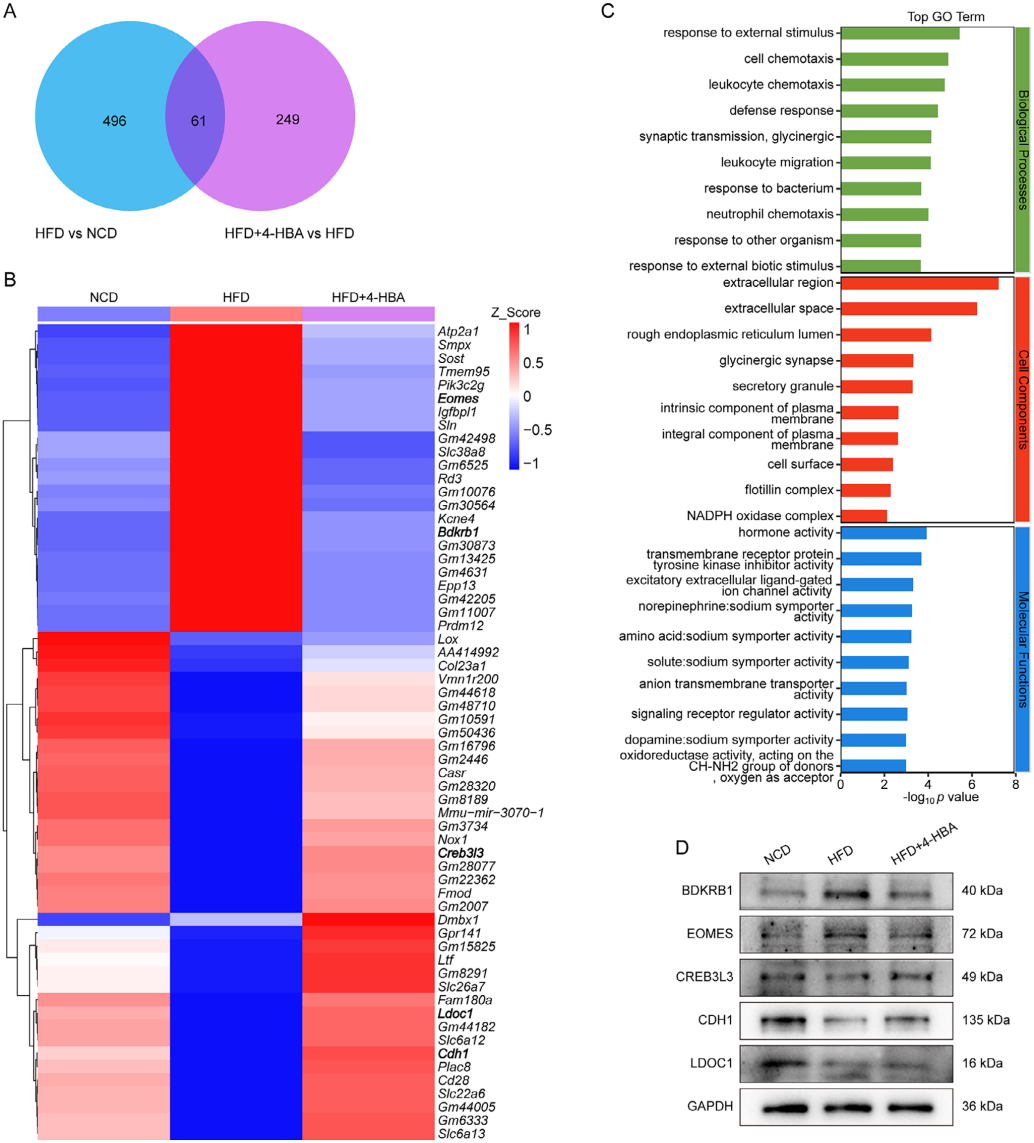
4‐HBA treatment altered the expression levels of inflammation‐related genes in the brains of HFD‐fed mice. (A) Venn diagram of DEGs among comparison groups. (B) Cluster heat map of shared DEGs across the three experimental mouse groups. (C) GO enrichment analysis of DEGs between the HFD+4‐HBA and HFD groups. (D) Representative Western blot images showed BDKRB1, EOMES, CREB3L3, CDH1, and LDOC1 levels in brain tissue from HFD‐fed mice treated with 4‐HBA (*n* = 3).

We performed an integrated analysis of the transcriptome and proteome on brain tissues from all experimental groups. RNA sequencing identified 557 significantly differentially expressed genes (DEGs) between the HFD and NCD groups, and 310 significant DEGs between the HFD+4‐HBA and HFD groups. Notably, 61 consensus DEGs showed expression changes across all three groups (Figure 4A,B; Table S2). Gene Ontology (GO) analysis revealed that DEGs identified between the HFD and HFD+4‐HBA groups were significantly enriched in biological processes, including response to external stimulus, cell chemotaxis, etc. (Figure 4C). Western blot analysis was performed to validate the protein products of key DEGs, including bradykinin receptor B1 (BDKRB1), eomesodermin (EOMES), cAMP‐responsive element‐binding protein 3‐like 3 (CREB3L3), cadherin 1 (CDH1), and leucine zipper downregulated in cancer 1 (LDOC1). 4‐HBA treatment downregulated BDKRB1 and EOMES in HFD mouse brains, while upregulating CREB3L3, CDH1, and LDOC1 with statistical significance (Figure 4D; Figure S7A–E).

**FIGURE 5:**
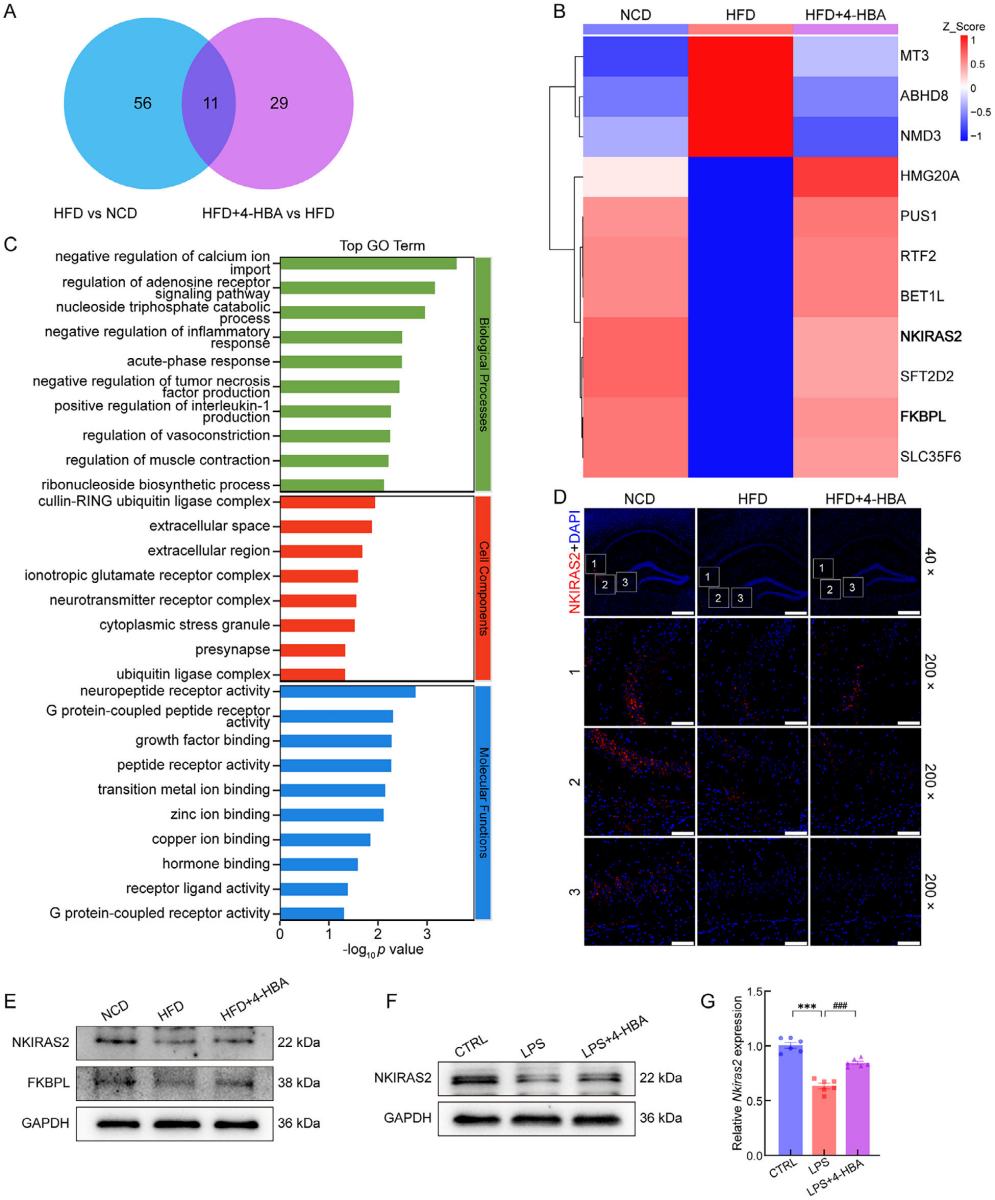
4‐HBA treatment altered the levels of inflammation‐related proteins in the brains of HFD‐fed mice. (A) The Venn diagram showed proteins differentially expressed among the comparison groups. (B) The cluster heat map showed shared differentially expressed proteins across the three mouse groups. (C) GO enrichment analysis of differentially expressed proteins between the HFD+4‐HBA and HFD groups. (D) Representative IF images showed NKIRAS2 expression in brain sections from NCD, HFD, and HFD+4‐HBA mice (*n* = 3), where red signals indicated NKIRAS2‐positive cells and blue signals represented DAPI‐stained nuclei (40×, scale bar: 500 µm; 200×, scale bar: 100 µm). (E) Representative Western blot images showed NKIRAS2 and FKBPL protein levels in brain tissue from HFD‐fed mice treated with 4‐HBA (*n* = 3). (F) Representative Western blot images showed NKIRAS2 protein levels in LPS‐stimulated BV2 cells treated with 4‐HBA (*n* = 3). (G) 4‐HBA treatment significantly upregulated *Nkiras2* mRNA expression in LPS‐stimulated BV2 cells (*p* < 0.0001; *n* = 6). Intergroup differences were analyzed using one‐way ANOVA with Tukey's post hoc test or Tamhane's T2 test. Data are expressed as mean ± SEM. ^***^ *p* < 0.001 versus CTRL BV2 cells; ^###^ *p* < 0.001 versus LPS‐stimulated BV2 cells.

According to the four‐dimensional data‐independent acquisition (4D‐DIA) quantitative proteome analysis, 67 proteins demonstrated significant differential expression when comparing HFD and NCD groups, 40 proteins showed an altered expression between HFD+4‐HBA and HFD groups, and 11 proteins exhibited expression changes shared among all three experimental groups (Figure 5A,B; Table S3). Based on GO enrichment analysis, differentially expressed proteins identified between the HFD and HFD+4‐HBA groups were significantly enriched in biological processes related to the negative regulation of inflammatory response and neurotransmitter receptor complex organization, etc. (Figure 5C). Among the differentially expressed proteins identified under HFD conditions, NKIRAS2 and FK506‐binding protein (FKBP)‐like (FKBPL) were prioritized because of their distinct modes of NF‐κB regulation. NKIRAS2 directly suppresses NF‐κB transcriptional activity [24], whereas the FKBP family indirectly modulates NF‐κB signaling through protein‐protein interactions [25]. 4‐HBA administration prevented the HFD‐induced downregulation of NKIRAS2 expression in the brain (Figure 5D; Figure S7F). Additionally, 4‐HBA significantly upregulated both NKIRAS2 and FKBPL protein levels in HFD mouse brains (Figure 5E; Figure S7G,H). In LPS‐stimulated BV2 cells, 4‐HBA significantly increased NKIRAS2 protein levels (Figure 5F; Figure S7I) and *Nkiras2* mRNA levels (Figure 5G).

**FIGURE 6:**
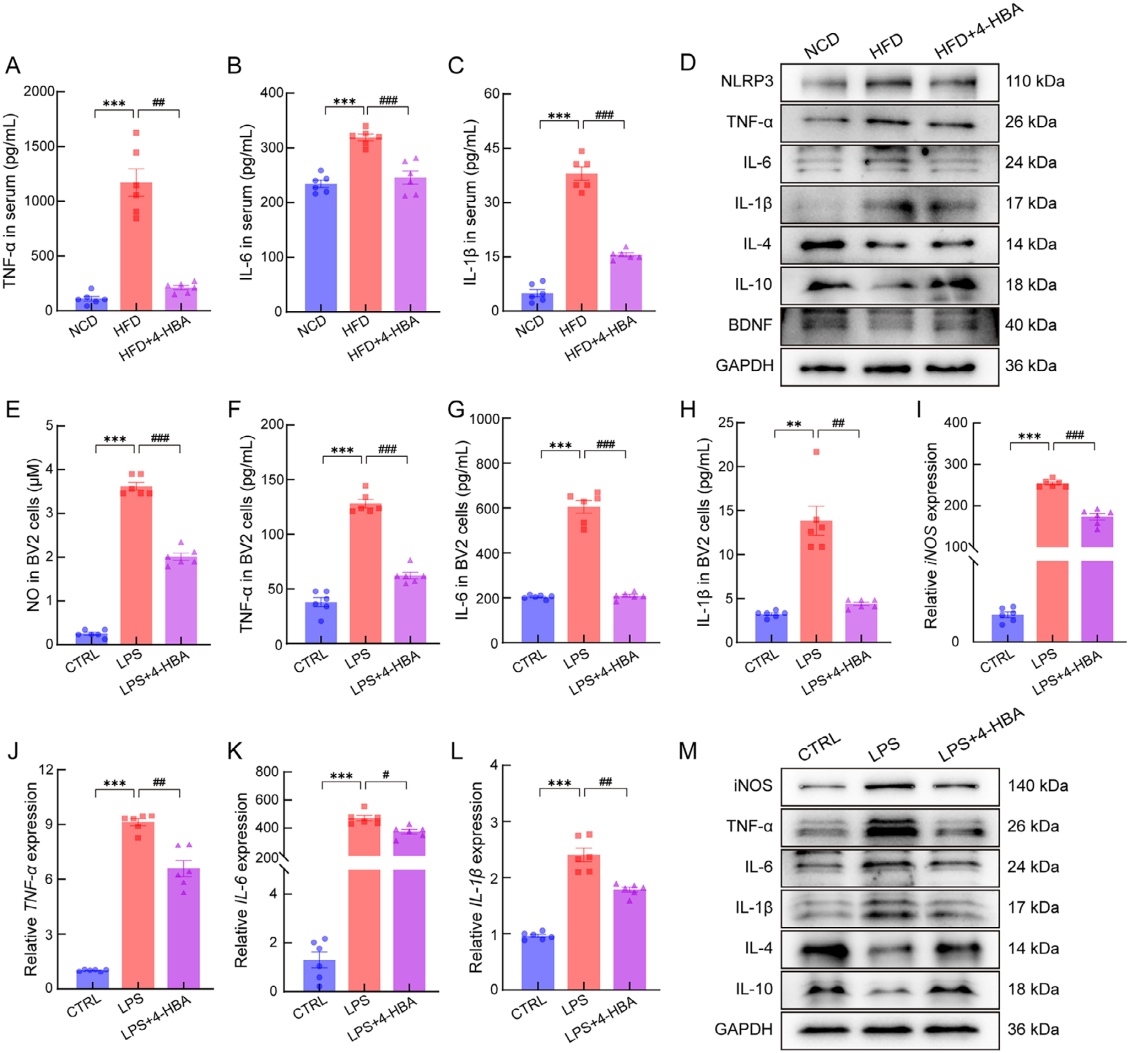
4‐HBA mitigated inflammatory responses in HFD‐fed mice and LPS‐stimulated BV2 cells. 4‐HBA treatment significantly reduced serum levels of (A) TNF‐α (*p* = 0.0016; *n* = 6), (B) IL‐6 (*p* = 0.0001; *n* = 6), and (C) IL‐1β (*p* = 0.0001; *n* = 6) in HFD‐fed mice. (D) Representative Western blot images of inflammatory and neurotrophic proteins in brain tissue (*n* = 3). In LPS‐stimulated BV2 cells, 4‐HBA significantly decreased supernatant levels of (E) NO (*p* < 0.0001; *n* = 6), (F) TNF‐α (*p* < 0.0001; *n* = 6), (G) IL‐6 (*p* = 0.0001; *n* = 6), and (H) IL‐1β (*p* = 0.0063; *n* = 6). 4‐HBA reduced mRNA expression of (I) *iNOS* (*p* = 0.0001; *n* = 6), (J) *TNF‐α* (*p* = 0.0037; *n* = 6), (K) *IL‐6* (*p* = 0.0100; *n* = 6), and (L) *IL‐1β* (*p* = 0.0079; *n* = 6). (M) Representative Western blot images of inflammatory proteins in LPS‐stimulated BV2 cells (*n* = 3). Intergroup differences were analyzed using one‐way ANOVA with Tukey's post hoc test or Tamhane's T2 test. Data are presented as mean ± SEM. ^***^ *p* < 0.001 versus NCD‐fed mice; ^##^ *p* < 0.01 and ^###^ *p* < 0.001 versus HFD‐fed mice for A–C. ^**^ *p* < 0.01 and ^***^ *p* < 0.001 versus CTRL BV2 cells; ^#^ *p* < 0.05, ^##^ *p* < 0.01, and ^###^ *p* < 0.001 versus LPS‐stimulated BV2 cells for E–L.

Four‐week 4‐HBA intervention achieved striking reductions of 81.9%, 22.9%, and 59.0% in serum levels of tumor necrosis factor‐α (TNF‐α), interleukin (IL)‐6, and IL‐1β, respectively, compared to vehicle‐treated HFD‐fed mice (Figure 6A–C). In HFD‐fed mice, 4‐HBA treatment significantly attenuated neuroinflammatory signaling. This was evidenced by a decrease in the protein expression levels of pro‐inflammatory mediators, including NLR family pyrin domain‐containing 3 (NLRP3), TNF‐α, IL‐6, and IL‐1β, alongside an upregulation of the anti‐inflammatory cytokines IL‐4 and IL‐10 (Figure 6D; Figure S8A–F). Furthermore, 4‐HBA upregulated brain‐derived neurotrophic factor (BDNF) levels in HFD‐fed mice, suggesting its capacity to ameliorate HFD‐associated neurotrophic impairment (Figure 6D; Figure S8G). As previously reported, LPS induces microglial activation and subsequent neuroinflammation [26]. Treatment with 25 µm 4‐HBA significantly attenuated LPS‐stimulated release of nitric oxide (NO), TNF‐α, IL‐6, and IL‐1β in cultured cells (Figure 6E–H). Quantitative real‐time PCR (qPCR) analysis revealed that 4‐HBA significantly inhibited LPS‐stimulated mRNA expression of inducible nitric oxide synthase (*iNOS*), *TNF‐α*, *IL‐6*, and *IL‐1β* in BV2 cells at 24 h post‐treatment (Figure 6I–L). Consistent with these findings, the protein levels of pro‐inflammatory mediators iNOS, TNF‐α, IL‐6, and IL‐1β were markedly suppressed, while the expression of the anti‐inflammatory cytokines IL‐4 and IL‐10 was significantly elevated (Figure 6M; Figure S8H–M).

To more closely approximate the in vivo microenvironment, we subsequently implemented a controlled stimulation system using BV2‐conditioned medium to assess how factors secreted by BV2 cells influence HT22 neuronal responses (Figure S9A). Direct stimulation of HT22 cells with LPS or 4‐HBA did not significantly alter cell viability or apoptosis (Figure S9B–D). LPS stimulation of HT22 cells markedly increased NO, TNF‐α, IL‐6, and IL‐1β levels in the culture medium, whereas 4‐HBA treatment restored these cytokines to baseline levels (Figure S9E–H). In contrast, conditioned medium from LPS‐stimulated BV2 cells markedly reduced HT22 cell viability and increased apoptotic cell death, whereas conditioned medium from BV2 cells co‐treated with LPS and 4‐HBA significantly restored cell survival and attenuated apoptosis (Figure S9I–K). Furthermore, conditioned medium from LPS‐stimulated BV2 cells induced a pronounced increase in NO, TNF‐α, IL‐6, and IL‐1β levels, whereas conditioned medium derived from BV2 cells co‐treated with LPS and 4‐HBA significantly attenuated this pro‐inflammatory response, restoring these mediators toward baseline levels (Figure S9L–O).

Given the robust anti‐inflammatory effects of 4‐HBA observed in both HFD‐fed mice and glial‐neuronal cellular systems, we next sought to determine whether peripherally administered 4‐HBA can cross the blood‐brain barrier and directly access the central nervous system. To investigate whether 4‐HBA can reach brain tissue, its concentrations in mouse plasma and brain were quantified at 0, 30, and 60 min post‐administration. LC‐MS/MS chromatograms showed clearly resolved parent ion peaks in both matrices, with retention times matching the authentic standard (Figure S10A,B). Quantitative analysis revealed detectable 4‐HBA in plasma and brain at all time points, showing a clear time‐dependent profile. Brain concentrations increased from 0.0072 ng/mg at 0 min to 0.0624 ng/mg at 30 min and subsequently decreased to 0.0221 ng/mg at 1 h (Figure S10C). Plasma concentrations peaked at 66.4680 ng/mL at 30 min and declined to 12.9553 ng/mL at 1 h (Figure S10D). This observation suggested that 4‐HBA had the ability to traverse the blood‐brain barrier, although its exposure within the brain was limited.

### 2.5 4‐HBA Ameliorated Neuroinflammation via the NKIRAS2/NF‐κB Pathway

**FIGURE 7:**
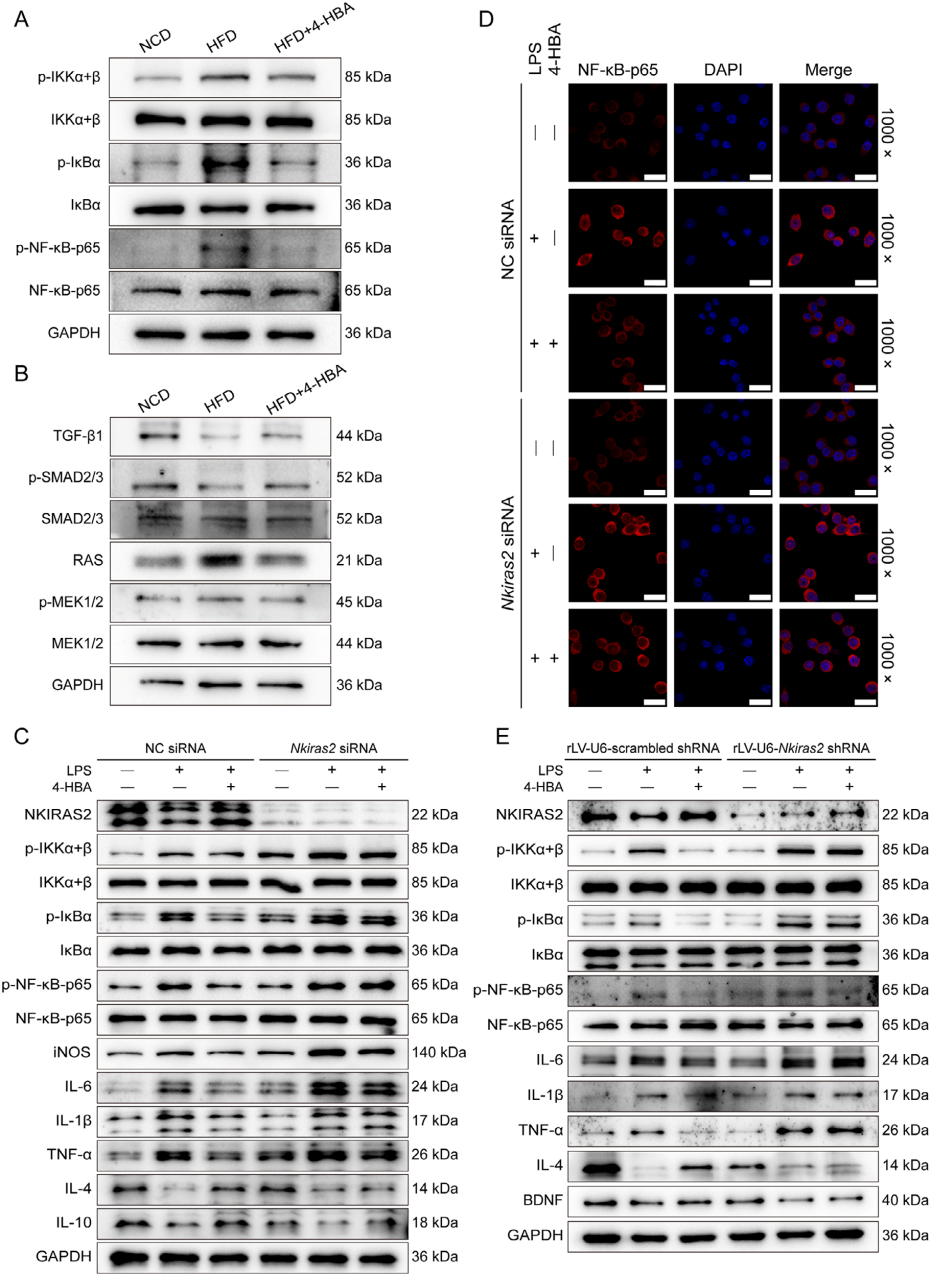
4‐HBA attenuated neuroinflammation through the NKIRAS2/NF‐κB pathway. (A) Representative Western blot images of phosphorylated NF‐κB pathway proteins in brain tissues from HFD‐fed mice treated with 4‐HBA (*n* = 3). (B) Representative Western blot images of TGF‐β/SMAD and RAS/MEK signaling pathway proteins in brain tissues from HFD‐fed mice treated with 4‐HBA (*n* = 3). (C) Representative Western blot images of NKIRAS2, inflammatory proteins, and phosphorylated NF‐κB pathway proteins in BV2 cells transfected with *Nkiras2* siRNA or NC siRNA (*n* = 3). (D) NF‐κB‐p65 nuclear translocation was visualized by confocal microscopy. Immunofluorescence signals were pseudocolored, with red indicating NF‐κB‐p65 and blue indicating DAPI‐stained nuclei (1000×, scale bar: 20 µm; *n* = 3). (E) Representative Western blot images of NKIRAS2, inflammatory and neurotrophic proteins, and phosphorylated NF‐κB pathway proteins in OBSCs transfected with *Nkiras2* shRNA or scrambled shRNA (*n* = 3).

Downregulation of NKIRAS2 amplifies inflammation‐induced NF‑κB activation [27], a pivotal signaling event that mediates immune‐inflammatory cascades and neurotoxic responses, thereby playing a central role in the pathophysiology of depression [28]. 4‐HBA‐treated HFD‐fed mice exhibited significant downregulation of phosphorylated inhibitor of κB (IκB) kinase (IKK)α+β, IκBα, and NF‐κB‐p65 compared to vehicle‐treated HFD‐fed mice (Figure 7A; Figure S11A–C). 4‐HBA‐treated HFD‐fed mice exhibited significantly elevated levels of TGF‐β1 and phosphorylated SMAD2/3, along with markedly reduced expression of RAS and phosphorylated MEK1/2 compared to vehicle‐treated HFD‐fed mice (Figure 7B; Figure S11D–G).

Since NKIRAS2 functions as an endogenous suppressor of NF‐κB signaling, we employed small interfering RNA (siRNA)‐mediated genetic silencing in BV2 cells to investigate its mechanistic requirement for 4‐HBA‐mediated neuroprotective effects. In LPS‐stimulated BV2 cells transfected with *Nkiras2* siRNA, 4‐HBA treatment neither increased NKIRAS2 expression nor inhibited the phosphorylation of IKKα+β, IκBα, or NF‐κB‐p65 compared with negative control (NC) siRNA‐treated cells (Figure 7C; Figure S12A–D). Critically, siRNA‐mediated *Nkiras2* depletion entirely abolished the anti‐inflammatory efficacy of 4‐HBA, as demonstrated by persistent upregulation of iNOS, IL‐6, IL‐1β, and TNF‐α, together with a pronounced suppression of IL‐4 and IL‐10 expression following 4‐HBA treatment (Figure 7C; Figure S12E–J). Our results suggested that NKIRAS2 mediated the effects of 4‐HBA on NF‐κB signaling. Moreover, 4‐HBA significantly inhibited LPS‐stimulated nuclear translocation of NF‐κB‐p65 in NC siRNA‐transfected BV2 cells, whereas *Nkiras2* knockdown substantially reduced this protective effect (Figure 7D).

To extend our mechanistic investigation beyond monocellular systems and into a more physiologically integrated context, we employed OBSCs (Figure S13A). In OBSCs, results paralleled those observed in *Nkiras2* siRNA‐transfected BV2 cells. Compared to scrambled shRNA‐treated OBSCs, transfection with shRNA targeting *Nkiras2* abolished the 4‐HBA induced upregulation of NKIRAS2 expression in the OBSCs (Figure 7E; Figure S13B). In scrambled shRNA‐treated OBSCs, LPS stimulation markedly increased the phosphorylation levels of IKKα+β, IκBα, and NF‐κB‐p65, whereas 4‐HBA treatment significantly attenuated these phosphorylation events (Figure 7E; Figure S13C–E). At the inflammatory level, LPS elicited a pronounced pro‐inflammatory response in scrambled shRNA‐treated OBSCs, as evidenced by elevated protein levels of IL‐6, IL‐1β, and TNF‐α, concomitant with a reduction in anti‐inflammatory cytokines. Notably, 4‐HBA treatment markedly attenuated this inflammatory response, as indicated by decreased expression of IL‐6, IL‐1β, and TNF‐α, together with increased levels of IL‐4 (Figure 7E; Figure S13F–I). Furthermore, 4‐HBA markedly increased BDNF levels, indicating an additional neuroprotective effect (Figure 7E; Figure S13J). In OBSCs transfected with *Nkiras2* shRNA, the protective effects of 4‐HBA were abolished. 4‐HBA failed to significantly reduce the phosphorylation of IKKα+β, IκBα, and NF‐κB‐p65, did not suppress the upregulation of pro‐inflammatory cytokines IL‐6, IL‐1β, and TNF‐α, and was unable to enhance the expression of IL‐4 and BDNF (Figure 7E; Figure S13C–J). These findings collectively confirmed that NKIRAS2 was an essential mediator through which 4‐HBA suppressed NF‐κB signaling and neuroinflammation, highlighting the pivotal role of NKIRAS2/NF‐κB regulation in mitigating hyperlipidemia‐associated depressive phenotypes.

As the primary immune cells of the central nervous system, microglia are crucial in mediating neuroinflammatory responses. To determine whether the anti‐inflammatory effects of 4‐HBA are accompanied by functional repolarization of microglia, we assessed the protein levels of key markers indicative of M1 and M2 polarization, including CD86 and CD206. 4‐HBA significantly modulated microglial polarization in the brains of HFD‐fed mice, reducing the expression of CD86 while enhancing CD206 (Figures S14A; Figure S15A,B). Consistent with these in vivo observations, 4‐HBA suppressed LPS‐induced upregulation of CD86 and promoted CD206 expression in LPS‐stimulated BV2 cells, reflecting a shift toward an anti‐inflammatory microglial phenotype. (Figures S14B; Figure S15C,D). To determine the critical role of NKIRAS2 in this phenotypic reprogramming, we performed RNA interference using siRNA in BV2 cells and lentivirus‐delivered shRNA in OBSCs to knock down *Nkiras2*. Notably, in both BV2 cells and OBSCs, 4‐HBA lost its ability to modulate the LPS‐induced expression of CD86 and CD206 (Figures S14C,D; Figure S15E–H). These findings indicated that NKIRAS2 was likely involved in the regulation of microglial phenotypic changes induced by 4‐HBA.

To assess the clinical relevance of our findings, we analyzed *NKIRAS2* gene expression in major depressive disorder (MDD) using the publicly available transcriptomic dataset GSE101521. Differential expression analysis revealed widespread transcriptional alterations in MDD patients compared with healthy controls (Figure S16A), with reduced *NKIRAS2* expression in MDD patients (Figure S16B). This observation was fully consistent with the diminished NKIRAS2 activity and the pro‐inflammatory phenotype observed in HFD‐fed mice. GO and KEGG pathway analyses further revealed that the DEGs were significantly enriched in pathways associated with inflammatory response regulation and NF‐κB signaling (Figure S16C,D). Collectively, these human disease data independently supported the dysregulation of neuroinflammation and NF‐κB signaling as pathological features of MDD, thereby providing strong translational evidence for our central mechanistic model in which 4‐HBA exerted antidepressant effects through modulation of the NKIRAS2/NF‐κB pathway.

## 3 Discussion

In this study, we employed integrated behavioral, molecular, and multi‐omics approaches to delineate the neuroinflammatory mechanisms linking hyperlipidemia to depression‐like phenotypes in HFD‐fed mice. Untargeted multi‐omics analysis suggested a neuroinflammatory cascade, and 4‐HBA emerged as a key metabolite associated with this process. Consistently, HFD induced depression‐like behaviors that were reversed by intraperitoneal 4‐HBA administration. At the molecular level, proteomic profiling identified a significant reduction of NKIRAS2 protein in the brains of HFD‐fed mice, and this decrease was restored after 4‐HBA treatment. Mechanistic perturbation further established causality. *Nkiras2* knockdown in BV2 cells and OBSCs enhanced NF‐κB activation and pro‐inflammatory cytokine production, abolishing the anti‐inflammatory effects of 4‐HBA. Together, these findings support a model in which 4‐HBA alleviates HFD‐induced depression‐like behaviors and neuroinflammation by upregulating NKIRAS2 and suppressing NF‐κB signaling.

The gut and the central nervous system establish a bidirectional communication network in which gut microbiota‐derived metabolites modulate neural function through neural, endocrine, and immune pathways [29]. Notably, *Akkermansia muciniphila* demonstrated significantly reduced relative abundance in chronic unpredictable mild stress depression model mice [30]. Bacterial treatment improved metabolic parameters in HFD‐fed mice [31] and suppressed LPS‐triggered TNF and NF‐κB activation, attenuating inflammatory cascades [32]. *A. muciniphila* increased gut butyrate levels [33], and this microbial metabolite ameliorated cognitive deficits in quinolinic acid‐exposed obese mice through neuroinflammatory suppression [34]. These results highlight the critical role of intestinal microbial communities and their metabolites in regulating central nervous system inflammation and neuronal activity. They also provide insight into HFD‐induced depression‐like phenotypes. We performed FMT using fresh and autoclaved HFD fecal slurry. Intriguingly, recipients of HFD fecal slurry and autoclaved HFD fecal slurry showed exacerbated depressive phenotypes, indicating that heat‐stable metabolites produced by the gut microbiota mediate these effects. Our findings suggested that 4‐HBA deficiency was associated with depression‐like phenotypes in HFD‐fed mice and may represent a potentially modifiable target for metabolic‐associated mood disorders. Although multiple metabolites were altered, this proof‐of‐concept study specifically focused on 4‐HBA due to its strong mechanistic relevance.

Hyperlipidemia promoted systemic inflammation by enhancing the release of pro‐inflammatory cytokines [35]. Under hyperlipidemic conditions, adipocytes activated NF‐κB signaling, inducing pro‐inflammatory gene expression [36]. Chronic low‐grade inflammation promoted depression‐like behaviors through the disruption of neurotransmitter synthesis and signaling [37] and the maladaptive remodeling of depression‐associated neural circuits. Blockade of inflammatory cytokines reduced depressive symptoms in both medically ill and medically healthy individuals with depression [38]. HFD feeding in the current study was associated with a pro‐inflammatory profile. Both serum and brain tissue exhibited elevated levels of pro‐inflammatory cytokines, along with reduced expression of anti‐inflammatory cytokines in the brain. These findings provided evidence for the coexistence of systemic and neurological inflammation under conditions of metabolic stress. Similarly, in BV2 cells stimulated with LPS in vitro, there was a strong induction of the pro‐inflammatory cytokines at both the mRNA and protein levels. In parallel, the anti‐inflammatory cytokines were significantly reduced in LPS‐challenged BV2 cells. 4‐HBA treatment significantly attenuated inflammatory responses in HFD‐fed mice and LPS‐stimulated BV2 cells, while concurrently improving metabolic parameters and depression‐like behaviors. To further clarify the cellular contributors to this inflammatory axis, we investigated whether microglia‐driven signals could relay the inflammatory stimuli to neuronal cells. Although BV2 cells were not fully equivalent to primary microglia, the conditioned‐medium system used here successfully recapitulated microglia‐neuron inflammatory interactions. These findings established neuroinflammation as the critical link between hyperlipidemia and depression.

Several key regulators identified in this study, including LDOC1, CDH1, FKBPL, and NKIRAS2, converge on the NF‐κB pathway to orchestrate neuroinflammatory responses. LDOC1 negatively regulates NF‐κB signaling by attenuating its transcriptional activity [39], and CDH1 expression displays an opposite trend relative to NF‐κB [40]. FKBPL modulates NF‐κB signaling indirectly via the glucocorticoid receptor pathway [25]. Notably, NKIRAS2 inhibits NF‐κB‐p65 phosphorylation [27], underscoring its pivotal role in mediating the anti‐inflammatory and potential antidepressant‐like effects of 4‐HBA. Collectively, these proteins form a regulatory network that fine‐tunes NF‐κB‐mediated neuroinflammation. Consistent with this notion, 4‐HBA treatment upregulated the expression of LDOC1, CDH1, FKBPL, and NKIRAS2. This suggests that 4‐HBA mitigated HFD‐induced neuroinflammation by reinforcing this NF‐κB‐centered anti‐inflammatory network. Together, the coordinated upregulation of these regulators may have influenced susceptibility to inflammation‐associated depressive phenotypes under HFD exposure.

Given that NKIRAS2 is a critical regulator of NF‐κB suppression, we performed targeted knockdown of *Nkiras2* to validate its indispensable role in this pathway. To avoid reliance on a single reductionist model and enhance translational relevance, we integrated cellular and tissue‐level experimental systems. BV2 cells are a thoroughly characterized and widely utilized in vitro model for investigating inflammatory signaling in microglia. This model is particularly well‐suited for studying LPS‐induced inflammatory responses that are dependent on NF‐κB activation [41]. OBSCs represent a physiologically relevant ex vivo platform that preserves the three‐dimensional cytoarchitecture of neural tissue and maintains intact cell‐cell and cell‐matrix interactions [42]. This system enables coordinated analysis of neurons, astrocytes, and microglia under controlled conditions, facilitating mechanistic studies of neuroinflammatory responses while reducing the reliance on whole‐animal experiments [43, 44]. Notably, knockdown of *Nkiras2* in LPS‐stimulated BV2 cells markedly exacerbated the inflammatory response and abolished the inhibitory effect of 4‐HBA on NF‐κB‐p65 nuclear translocation. Consistent with these cellular observations, lentivirus‐mediated suppression of NKIRAS2 in OBSCs produced a highly concordant phenotype. *Nkiras2* depletion abolished the suppressive effect of 4‐HBA on NF‐κB activation and pro‐inflammatory cytokine production. It also prevented the restoration of anti‐inflammatory and neurotrophic signaling. Critically, these findings extended the functional requirement of NKIRAS2 from reductionist microglial models to an intact brain tissue context that preserved native cytoarchitecture and intercellular interactions. We indicated that NKIRAS2 acted as more than a cell‐autonomous regulator of NF‐κB signaling. It also functioned as an essential mediator of the anti‐inflammatory and neuroprotective effects of 4‐HBA within the neural microenvironment. Collectively, our data supported a model in which 4‐HBA exerted its pharmacological actions through the upregulation of NKIRAS2. This axis was essential for constraining NF‐κB signaling and the downstream neuroinflammatory cascade.

Our findings indicated that 4‐HBA can alleviate depressive‐like phenotypes by attenuating neuroinflammation through the NKIRAS2/NF‐κB pathway. Beyond the classical pro‐inflammatory cytokine cascade, neuroinflammation is also characterized by a dynamic shift in microglial activation states [44]. Notably, in HFD‐fed mice and LPS‐stimulated BV2 cells, 4‐HBA treatment suppressed a broad spectrum of inflammatory mediators and modulated specific activation markers, reducing the M1‐associated marker CD86 while increasing the M2‐associated marker CD206. This shift suggested that 4‐HBA not only dampened inflammatory output but may also have actively driven functional repolarization of microglia toward a more homeostatic, anti‐inflammatory phenotype. Knockdown of *Nkiras2* abolished the effects of 4‐HBA on CD86 and CD206 expression. These findings suggest that the NKIRAS2/NF‐κB pathway functions as a regulatory axis involved in both inflammatory responses and microglial phenotypic transitions. This dual function likely underlies the marked improvement of depression‐like behaviors observed in HFD‐fed mice, underscoring a promising therapeutic strategy targeting the neuroimmune interface in metabolism‐associated depression.

We identified 4‐HBA as a suppressor of neuroinflammation acting through the NKIRAS2/NF‐κB pathway. This discovery revealed a novel therapeutic mechanism for depression that complements existing anti‐inflammatory strategies. The tetracycline antibiotic minocycline exhibits neuroimmune‐modulating properties and demonstrates antidepressant efficacy in clinical trials [45, 46]. Evidence indicates its therapeutic effects involve suppression of downstream inflammatory mediators, with enhanced benefits observed in patients exhibiting elevated inflammation [47]. Similarly, the cyclooxygenase‐2 inhibitor celecoxib ameliorates depressive‐like phenotypes by inhibiting NF‐κB signaling and reducing pro‐inflammatory markers [48]. Mechanistically, 4‐HBA enhanced cellular sensitivity to NF‐κB inhibition by upregulating NKIRAS2, potentially promoting synergistic effects with these agents. Notably, chronic intraperitoneal administration of 4‐HBA did not cause detectable histopathological alterations in major organs, indicating no overt toxicity. Human transcriptomic evidence of immune and NF‐κB pathway dysregulation in MDD further substantiated the translational relevance of our findings.

Although this study systematically delineated the mechanism by which 4‐HBA alleviated neuroinflammation and ameliorated metabolic depression via the NKIRAS2/NF‐κB pathway, several important limitations should be acknowledged. Caution is warranted when extrapolating findings from animal models. This study exclusively employed male C57BL/6J mice, and the HFD model cannot fully recapitulate the complexity of human metabolic depression comorbidity, including genetic background, sex differences, and psychosocial factors. In addition, given the substantial interspecies differences in gut microbiota composition and metabolic pathways, the 4‐HBA‐related metabolic features observed in rodents require further validation in humans. Although our pharmacokinetic analysis confirms brain penetration of 4‑HBA, the limited sampling time points preclude a full characterization of central pharmacokinetic parameters. Future studies with more extensive sampling will be required to comprehensively define the relationship between central exposure and pharmacodynamic effects. Moreover, while our study identified NKIRAS2 as a central mediator of 4‐HBA's anti‐inflammatory action, it likely functioned within a broader signaling network. This study did not systematically assess oxidative stress markers or antioxidant signaling pathways. Although a comprehensive evaluation of these pathways was beyond the scope of the present study, their investigation represents an important direction for future research. Such studies will be crucial to disentangle NKIRAS2‐specific effects from more general anti‐inflammatory and redox‐modulating mechanisms.

## 4 Conclusion

4‐HBA was found to be a key regulatory factor in the HFD‐induced metabolic dysregulation, depression‐like behaviors, and neuroinflammation. These findings reveal molecular pathways that link hyperlipidemia with depressive pathogenesis, advancing our understanding of these bidirectional relationships. Mechanistically, 4‐HBA alleviates hyperlipidemia‐associated depression‐like phenotypes by modulating neuroinflammation through the NKIRAS2/NF‐κB pathway. To advance the clinical application of 4‐HBA in the future, translational research will be essential.

## 5 Experimental Section

### 5.1 Lentiviral Production and Titration

Lentiviral vectors were constructed, and the corresponding plasmids were prepared using standard molecular cloning and large‐scale purification techniques. 293T cells were plated in 10 cm dishes and allowed to reach 70%–80% confluence by the following day. One hour before transfection, the culture medium was replaced with 10 mL of Opti‐MEM. A transfection mixture was then prepared by gently combining 32 µg of plasmid DNA with the appropriate volume of transfection reagent. The plasmid mixture consisted of packaging and transfer plasmids at a 1:1 ratio. After a 20 min incubation at room temperature, the DNA‐transfection reagent complexes were added dropwise to the cells. Viral supernatants were harvested at 48 and 72 h post‐transfection. Viral pellets were resuspended in DPBS, aliquoted, and stored at −80°C until use. 293T cells were infected with serially diluted viral preparations, and genomic DNA was extracted 72–96 h after infection. VCN was determined by qPCR targeting the WPRE sequence, with infectious titers expressed as transducing units per milliliter (TU/mL).

### 5.2 Animal Models and Treatment

The experimental protocols involving animals were performed in compliance with ARRIVE guidelines and received ethical approval from the Institutional Animal Care and Use Committees, Jilin Agricultural University (Approval No. 20230418001) and Jilin University (Approval Nos. SY202310024, SY202511015, and SY202511042). Male C57BL/6J mice (Liaoning Changsheng Biotechnology, SCXK[Liao]2020‐0001) were housed in specific pathogen‐free facilities under controlled conditions: 12‐h light/dark cycles, 23°C ± 1°C temperature, and 40%–60% relative humidity.

The 11–12‐week‐old male C57BL/6J mice were randomly assigned to two experimental groups (*n* = 10 per group). The NCD group was fed a normal chow diet (D12450B, 10% kcal fat, Liaoning Changsheng Biotechnology, Benxi, China), while the HFD group received a high‐fat diet (D12492, 60% kcal fat, Xiao Shu You Tai Biotechnology, Beijing, China) for 17 consecutive weeks. Weekly fecal collection commenced during the last 5 weeks of dietary treatment. Depression‐related behavioral assessments were conducted in the terminal week of the study period (Figure 1A).

Male C57BL/6J mice (4–5 weeks old) received a 4‐week gut microbiota depletion protocol through administration of a broad‐spectrum antibiotic cocktail (ABX; 1 g/L metronidazole, 0.5 g/L vancomycin, 1 g/L ampicillin, and 1 g/L neomycin) in drinking water. After antibiotic treatment, mice were randomly allocated to three groups receiving FMT, with donor slurry (300 µL of 100 mg/mL in 10% glycerol/saline) administered every other day for 2 weeks. The NF group received NCD fecal slurry (*n* = 6), the HF group received HFD fecal slurry (*n* = 6), and the AHF group received autoclaved HFD fecal slurry (121°C for 15 min; *n* = 6). All mice received a normal chow diet, with behavioral tests performed in the final experimental week (Figure 2A).

To evaluate the effects of 4‐HBA on hyperlipidemia and depression‐like phenotypes, 30 male C57BL/6J mice (11–12 weeks old) were assigned to either an NCD group (*n* = 10) or an HFD group (*n* = 20) for a 12‐week induction phase. Following this induction period, the HFD group was subdivided to receive either normal saline (10 mL/kg/day, i.p.) (*n* = 10) or 4‐HBA (40 mg/kg/day, i.p.) (*n* = 10) (S25591, Shanghai Yuanye, Shanghai, China) for 4 weeks, while the NCD group continued saline administration (10 mL/kg/day, i.p.). Following 4 weeks of continuous 4‐HBA intervention, depression‐like behaviors were systematically evaluated through behavioral tests (Figure 3A).

4‐HBA's long‐term safety was studied for possible histopathological effects it might cause on the main organs if one is continuously exposed. The mice were randomly divided into two groups: a control group (*n* = 6) receiving intraperitoneal saline and a treatment group (*n* = 6) receiving 4‐HBA (40 mg/kg, i.p.). All animals received treatment for 6 months straight without stopping. Throughout the experiment, all animals were housed under standard laboratory conditions and fed a regular diet.

To assess the capacity of 4‐HBA to penetrate the blood‐brain barrier, a single intraperitoneal injection of 40 mg/kg 4‐HBA was administered to male C57BL/6J mice, aged 6–8 weeks. Brain tissue and plasma samples were collected at 0, 30, and 60 min post‐injection for quantification by LC‐MS/MS (*n* = 6).

At the study endpoint, terminal blood collection was performed via tail vein puncture immediately preceding humane euthanasia through controlled CO_2_ asphyxiation. Following this procedure, multiple organs, pWAT, eWAT, BAT, and iWAT were carefully dissected, with each specimen being precisely weighed. A subset of tissue samples was cryopreserved in liquid nitrogen and stored at −80°C, while corresponding aliquots were fixed in 4% neutral buffered paraformaldehyde (BL539A, Biosharp, Hefei, China) for 24 h for histological assessment.

### 5.3 Brain Slice Culture

Mice were euthanized under sterile conditions and immediately decapitated. The brains were rapidly removed and immersed in chilled dissection medium composed of Hibernate‐A medium (A1247501, Thermo Fisher Scientific, Waltham, MA, USA), supplemented with 2% B‐27 (PB180637, Procell, Wuhan, China), 2 mm L‐glutamine (PB180420, Procell, Wuhan, China), and 1% penicillin‐streptomycin‐gentamicin. All procedures were conducted under aseptic conditions to maintain tissue viability. Mouse brains were sectioned into 300 µm slices in freshly prepared dissection medium. Individual slices were carefully transferred onto semiporous membrane inserts (PICMORG50, Millipore, Burlington, MA, USA) placed in culture plates. OBSCs were cultured using the interface method at a pH of 7.2 and incubated at 37°C in a humidified environment with 5% CO_2_. The culture medium consisted of Neurobasal‐A medium (PM151236, Procell, Wuhan, China) supplemented with 2% B‐27, 2 mm L‐glutamine, and 1% penicillin‐streptomycin‐gentamicin, with the optional addition of a trace amount of horse serum based on the condition of the brain slices. The culture medium was replaced on the day following slice preparation and subsequently refreshed every other day. On day 3 in vitro, a diluted viral vector solution at a concentration of 2.5 × 10^8^ TU/mL in PBS was carefully applied directly onto each slice in a volume of 40 µL. After 24 h of incubation, the slices were washed to remove residual virus and transferred to fresh culture plates. Cultures were maintained for 7–14 days post‐transduction, after which slices were treated with LPS (1 µg/mL) or 4‑HBA (25 µm). After pharmacological stimulation, all slices were rapidly frozen in liquid nitrogen.

### 5.4 SPT

A reduction in sucrose preference (%) is widely regarded as a validated indicator of anhedonia in rodent models. The experimental protocol comprised four sequential phases. During the habituation phase, individually housed mice received two identical 1% sucrose bottles symmetrically positioned for a 24‐h period. During the training phase, one sucrose bottle was replaced with ultrapure water for 24 h, with bottle positions switched every 12 h to control side preference. Prior to testing, animals underwent a 24‐h food and water deprivation period. The final test phase involved presentation of pre‐weighed bottles containing either 1% sucrose or water, positioned at equal heights for 12 h, with positions swapped at 6 h to control for spatial bias. Sucrose preference (%) was calculated as: [(sucrose consumption) / (water consumption + sucrose consumption)] × 100%.

### 5.5 TST

Each mouse was hung on the distal one‐third of its tail with low‐allergy tape, keeping its head down at a height of 50 cm from the ground. All 6‐min sessions were recorded for later behavioral scoring. To avoid stress‐induced bias, each mouse was allowed 2 min before collecting data, with only the remaining 4 min used for analysis. Immobility time refers to the period during which the mice stop engaging in any struggling behaviors and remain in a standing position without movement. The immobility time was automatically recorded and analyzed by DigBehv Animal Behavior Analysis System (JLBehv‐FSG‐4, Shanghai Jiliang, Shanghai, China).

### 5.6 OFT

Mice in the first two experimental batches were tested using an open field system (OFT‐100, Chengdu Techman, Chengdu, China), whereas mice in the final batch were assessed with a different instrument (ZS_ZFT, Beijing Zhongshi Dichuang Technology, Beijing, China). During the 5‐min test session, the following parameters were automatically recorded using video‐tracking software: center zone time, number of rearings, total distance moved, and central zone entries. Animals were habituated to the testing apparatus for 1 min before behavioral assessment. This procedure minimized stress responses to the novel environment.

### 5.7 FST

To evaluate a depression‐like behavioral state, the FST was conducted in a transparent plexiglass cylinder filled with 25 ± 1°C purified water, which was replaced between trials to maintain hygiene. Each mouse underwent a 6‐min trial during which immobility time was quantified using automated tracking software. Mice underwent a 2‐min acclimation in water before testing to minimize stress‐induced bias. Data from the last  4 min were used for behavioral analysis. Immobility duration was quantified automatically using the DigBehv Animal Behavior Analysis System (JLBehv‐FSG‐4, Shanghai Jiliang, Shanghai, China). A mouse was considered immobile during periods in which it ceased all struggling movements while maintaining an upright posture.

### 5.8 Blinding and Randomization

All behavior assessments, including SPT, FST, TST, and OFT, were done properly with good blinding and randomization. Experimental animals were randomly assigned to treatment groups. Investigators involved in behavioral testing and data analysis were blinded to the group assignments throughout the study. To make the measurement more objective, two experienced researchers who had received proper training would be responsible for conducting behavioral quantification separately. Their ability to work together had already been tested before they started collecting real data.

### 5.9 Untargeted Metabolomics Analysis

Untargeted metabolomic analysis was conducted on cerebral tissue specimens (*n* = 3 per group) from NF and AHF groups at Personalbio Technology Co., Ltd. Raw data were converted using ProteoWizard (v3.0.8789) and subsequently analyzed in XCMS (v3.12.0) for peak detection, retention time alignment, and extraction of peak areas. All samples belonged to the same biological category. During preprocessing, peak alignment and quality control (QC)‐based filtering were performed to ensure data consistency. Feature tables underwent metabolite identification and quantification, followed by preprocessing, including filtering, normalization, and missing‐value imputation. The quality of the data was assessed through QC evaluations. Features with a relative standard deviation (RSD) exceeding 30% in the QC samples were excluded to improve the reliability and consistency of the dataset. Metabolite identification was conducted by comparing retention times and accurate masses, with a tolerance of less than 10 ppm. All metabolite identifications were made by comparing experimental MS/MS spectra and collision energies against both an in‐house spectral library and major public databases, achieving confidence levels of 2 or above. Metabolites exhibiting significantly differential abundance were identified based on the following criteria: variable importance in projection (VIP) > 1 and *p* value < 0.05. Pathway enrichment analysis was performed using the clusterProfiler package (v4.6.0) with Fisher's exact test against the KEGG database.

### 5.10 Transcriptomic Analysis

Brain samples were analyzed by transcriptomics at APTBIO. Total RNA from brain tissues (*n* = 3 per group) was extracted using TRIzol and evaluated with NanoDrop ND‐2000 and Bioanalyzer 4150. Libraries prepared with the ABclonal mRNA‐seq kit were sequenced on Illumina NovaSeq 6000 and MGISEQ‐T7 platforms to ensure technical reproducibility. Raw reads were subjected to quality filtering and then aligned to the mouse reference genome. Cross‐platform analytical consistency was preserved by implementing a standardized normalization and statistical framework. Comparative expression profiling was performed across defined contrasts (HFD vs. NCD and HFD+4‐HBA vs. HFD) using featureCounts for counting. Differential expression analysis was then conducted using DESeq2 with standard parameters. The DESeq2 algorithm incorporates median‐of‐ratios normalization, and significance thresholds of absolute log_2_ fold change (FC) greater than 1 and a *p* value less than 0.05 were applied for identifying significantly regulated transcripts. GO enrichment analysis was performed using the clusterProfiler package.

### 5.11 4D‐DIA Quantitative Proteome Analysis

Brain tissues underwent quantitative proteomic analysis via an advanced 4D‐DIA mass spectrometry platform at PTM BioLab, based on the DIA‐PASEF methodology (*n* = 3 per group). After separation using ultra‐high‐performance liquid chromatography on a NanoElute system, the peptide mixtures underwent mobility‐enhanced analysis on a timsTOF Pro instrument. All acquired spectral data were processed with the DIA‐NN platform (version 1.8) against the Mus_musculus_10090_SP_20231220.fasta database comprising 17 191 protein entries. Spectral library generation employed deep learning‐predicted spectra with decoy databases, maintaining a false discovery rate threshold below 1%. Quantitative proteomic profiles were derived from normalized intensity values processed via mean‐centering transformation. Reproducibility across technical replicates was confirmed through principal component analysis. To achieve an appropriate balance between false positive control and detection sensitivity, proteins were considered differentially expressed if they met both of the following criteria: a nominal *p* value < 0.05 and FC either greater than 1.5 or less than 1/1.5. Functional characterization was achieved through Gene Ontology classification using EggNOG‐mapper annotation.

### 5.12 Histopathological Analysis

Following established protocols from our previous work [49], we conducted histological analyses on multiple tissue types. Paraffin‐embedded samples of adipose depots (eWAT, iWAT, pWAT, BAT) and visceral organs (brain, heart, spleen, kidney) were processed into 5‐µm paraffin sections for H&E staining to evaluate histological architecture. For hepatic lipid evaluation, 10‐µm cryosections of frozen liver tissue were prepared, then processed for both Oil Red O and H&E staining. All imaging was performed using a Nikon Eclipse Ci‐L microscope (Eclipse Ci‐L, Nikon Corporation, Tokyo, Japan) or a research‐grade whole‐slide scanning system (VS4000, Olympus, Tokyo, Japan).

### 5.13 TEM

Small tissue samples (≤1 mm^3^) were initially fixed in chilled 4°C fixative solution (B0012, BaiQianDu Biotechnology, Wuhan, China) for 24 h, then washed three times (15 min each) with 0.1 m PBS. Post fixation was performed with 1% osmic acid in PBS (20°C, 2 h), and samples were re‐rinsed in PBS. Dehydration proceeded through a graded ethanol (10009218, Sinopharm Chemical Reagent, Shanghai, China) series, after which tissues were infiltrated sequentially with acetone, EPON 812 resin (1:1 v/v) overnight, followed by pure EPON 812 resin (90529774, SPI Supplies, West Chester, PA, USA) for an additional 24 h. Polymerization was performed at 60°C over a 48‐h period. Ultramicrotomy‐generated 70‐nm sections underwent dual staining, first with 2% uranyl acetate (15 min) and subsequently with Reynold's lead citrate solution (15 min). Following natural evaporation at room temperature, the specimens were imaged with a Tecnai transmission electron microscope (Tecnai G2 20 TWIN, FEI, Hillsboro, OR, USA).

### 5.14 IF analysis

All procedures followed our previous work [50]. Brain specimens underwent fixation in 4% paraformaldehyde (4°C, 24 h), then were processed through paraffin removal and heat‐induced epitope retrieval using EDTA buffer (pH 8.0). To quench endogenous peroxidase activity, slide‐mounted sections received 3% H_2_O_2_ (25 min, 25°C, dark conditions). After drying, tissue boundaries were delineated with hydrophobic markers. Background blocking was achieved with 10% goat serum (30 min, 25°C) prior to 4°C overnight exposure to primary antibodies (Table S4). Following phosphate‐buffered saline rinses, HRP‐conjugated secondary antibodies (Table S4) were incubated (50 min, 25°C, dark conditions), followed by tyramide‐fluorophore signal amplification (20 min). Nuclear visualization was accomplished with DAPI staining (10 min) before antifade mounting and microscopic analysis.

### 5.15 Cytokine Detection

We measured the levels of pro‐inflammatory cytokines in the serum and culture supernatants, such as IL‐1β (E‐EL‐M0037, Elabscience, Wuhan, China), IL‐6 (E‐EL‐M0044, Elabscience, Wuhan, China), and TNF‐α (E‐EL‐M3063, Elabscience, Wuhan, China). Enzymatic colorimetric assay kits were employed to measure serum levels of lipid factors in mice, using dedicated TC detection kit (A111‐1‐1, Nanjing Jiancheng Bioengineering Institute, Nanjing, China), the TG detection kit (A110‐1‐1, Nanjing Jiancheng Bioengineering Institute, Nanjing, China), the LDL‐C detection kit (A113‐1‐1, Nanjing Jiancheng Bioengineering Institute, Nanjing, China), and the HDL‐C detection kit (A112‐1‐1, Nanjing Jiancheng Bioengineering Institute, Nanjing, China).

### 5.16 Western Blot

Cerebral tissues and BV2 cells were lysed in RIPA buffer containing 1% lytic protease and phosphatase inhibitor cocktail on ice, followed by centrifugation to obtain protein extracts. Total protein content was quantified using the BCA assay. The 40 µg aliquots of each sample were resolved on SDS‐PAGE and electrotransferred to PVDF membranes (10600023, Cytiva, Shanghai, China). After blocking, membranes were incubated with primary antibodies (Table S4) and corresponding HRP‐conjugated secondary antibodies (Table S4). Protein bands were visualized using ECL substrate (K1231, APExBIO, Houston, TX, USA) on an imaging system (Tanon‐5200, Tanon, Shanghai, China).

### 5.17 LC‐MS/MS

Quantitative analysis of 4‐HBA in mouse brain tissue and plasma was performed using an LC‐MS/MS system. Plasma samples measuring 50 µL were treated with three volumes of methanol for protein precipitation and then centrifuged. Brain tissues were homogenized in methanol. Ultrasonic extraction and centrifugation were then performed, and the resulting supernatants were analyzed by LC‐MS/MS. A reference standard of 4‐HBA (R011776, RHAWN, Shanghai, China), with a purity of 98%, was dissolved in methanol to prepare a stock solution. This stock was then serially diluted with water to produce calibration standards at specified concentrations. Calibration curves were constructed using these solvent‐based standards. All measured plasma concentrations were corrected using the corresponding dilution factor. Chromatographic separation was performed using a Thermo Scientific Vanquish ultra‐high‐performance liquid chromatography system. The mobile phase consisted of water as solvent A and acetonitrile as solvent B, mixed in a volume ratio of 80:20. The flow rate was set at 0.3 mL/min, and a sample volume of 1 µL was injected. Mass spectrometric analysis was performed using a Quantis triple quadrupole mass spectrometer (Thermo Fisher Scientific, Waltham, MA, USA) equipped with an electrospray ionization source operating in negative ion mode. Data were acquired and quantified via TraceFinder 4.1 General Quan software (Thermo Fisher Scientific, Waltham, MA, USA) in multiple reaction monitoring mode. Ion source parameters were optimized with electrospray voltage 2.5 kV, nebulizer temperature 350°C, ion transfer tube temperature 325°C, sheath gas flow 50 Arb, and auxiliary gas flow 10 Arb.

### 5.18 Cell Culture and Treatment

BV2 cells (T9077, Applied Biological Materials (abm), Vancouver, Canada) and HT22 cells (iCell‐m020, Cellverse, Shanghai, China) were cultured in Dulbecco's Modified Eagle Medium containing 10% FBS and 1% penicillin‐streptomycin‐gentamicin solution. The cells were incubated under standard conditions throughout the experimental period. BV2 cells were pretreated with 25 µm 4‐HBA for 1 h and subsequently stimulated with 1 µg/mL LPS (S1732, Beyotime, Shanghai, China) for 24 h. After incubation, the BV2 culture medium was centrifuged to obtain cell‐free conditioned medium. The conditioned medium was used to replace the complete DMEM previously applied to HT22 cells. HT22 cell viability was assessed using the CCK‐8 assay and Annexin V‐FITC/PI staining.

### 5.19 Cell Viability Assay

Cell viability was assessed using the CCK‐8 assay (K1081, APExBIO, Houston, TX, USA). HT22 cells were treated with LPS, 4‐HBA, or BV2‐conditioned medium for 24 h, with six replicate wells per group. The following day, the culture medium was replaced with fresh medium supplemented with CCK‐8 reagent. The absorbance at 450 nm was recorded using a microplate reader.

### 5.20 Apoptosis Assay

Apoptosis in HT22 cells was evaluated using an Annexin V‐FITC/PI apoptosis detection kit (CX006S, EpiZyme, Shanghai, China). Both adherent and floating cells were collected, washed with PBS, and centrifuged. Cells were resuspended in 100 µL binding buffer and then stained with 5 µL AV‐FITC and 5 µL PI. After incubation for 15 min in the dark, apoptosis was quantified using a flow cytometer (FongCyte C3140, Beijing Challen Biotechnology, Shanghai, China).

### 5.21 Griess Assay

Nitrite levels in culture media were measured using the Griess assay as an indicator of NO production. After pelleting cellular debris via centrifugation, 50 µL supernatant samples were dispensed into a 96‐well plate and combined with an equal volume of Griess reagents I and II (S0021, Beyotime, Shanghai, China). Optical density measurements were then performed at 540 nm wavelength using a microplate spectrophotometer.

### 5.22 RNA Interference (RNAi) of *Nkiras2*

To achieve *Nkiras2* knockdown, BV2 cells were transfected with 120 nM *Nkiras2* siRNA, with NC siRNA (120 nM) serving as a negative control. Following a 24‐h transfection period, cells were pretreated with 25 µm 4‐HBA for 1 h before being exposed to LPS (1 µg/mL, 24 h). All transfections were performed using GP‐transfect‐Mate (G04008, GenePharma, Shanghai, China). The siRNA duplexes targeting *Nkiras2* and NC were synthesized by Sangon Biotech with the following sequences: *Nkiras2*‐sense, 5ʹ‐ UCGAGCUGCUCAAGAAGGA/dT/dT/‐3ʹ; *Nkiras2*‐antisense, 5ʹ‐ UCCUUCUUGAGCAGCUCGA/dT/dT/‐3ʹ; NC‐sense, 5ʹ‐ UUCUCCGAACGUGUCACGU/dT/dT/‐3ʹ; NC‐antisense, 5ʹ‐ ACGUGACACGUUCGGAGAA/dT/dT/ ‐3ʹ.

### 5.23 qPCR

Cellular RNA was purified from lysates employing silica‐membrane spin columns (K0731, Thermo Fisher Scientific, Waltham, MA, USA), with subsequent complementary DNA synthesis performed using PrimeScript RT reagents (CW3371, CWBIO, Jiangsu, China). Real‐time quantitative PCR amplification was carried out on a Bio‐Rad CFX96 platform with SYBR Green chemistry (CW3360, CWBIO, Jiangsu, China), with all reactions performed in technical triplicate. Gene expression quantification was determined through the 2^^(‐ΔΔCt)^ method, using *GAPDH* as the housekeeping gene. The oligonucleotide primers used in this study were commercially synthesized by Sangon Biotech, with complete sequence information provided in Table S5.

### 5.24 Determination of NF‐κB Activity

BV2 cells underwent 24 h transfection with either NC siRNA or *Nkiras2* siRNA. After pretreatment with 4‐HBA (25 µm, 1 h), cultures were stimulated with LPS (1 µg/mL, 24 h). NF‐κB‐p65 nuclear translocation was assessed using a commercial assay kit (SN368, Beyotime, Shanghai, China), with visualization conducted via Olympus FV4000 confocal microscopy (FV4000, Olympus, Tokyo, Japan).

### 5.25 Transcriptomic Analysis in Human Depression

The dataset GSE101521 comprises postmortem brain samples from suicide MDD patients, non‐suicide MDD patients, and non‐depressed controls. Following quality control, differential expression analysis was performed using DESeq2 with thresholds of |log_2_ fold change| > 0.1 and *p* < 0.05. To investigate the potential biological functions of the DEGs, we performed GO and KEGG pathway analyses. The GO analysis encompassed categories such as biological processes, cellular components, and molecular functions, and was conducted using the enrichGO function from the clusterProfiler package. KEGG pathway enrichment was subsequently performed using the enrichKEGG function within the same package to identify relevant signaling pathways. Gene annotation was provided by the org.Hs.eg.db package in R.

### 5.26 Statistical Analysis

Statistical analyses and data visualization were conducted using GraphPad Prism 9, SPSS Statistics 26, and R software (v.4.5.2) in conjunction with RStudio (v.2025.9.2.0). Experimental results are expressed as mean ± SEM. Prior to inferential testing, data distribution assumptions were evaluated. For pairwise comparisons, normally distributed data were analyzed using a two‐tailed unpaired Student's *t*‐test. For comparisons involving multiple groups, normality was initially assessed using the Shapiro–Wilk test, and the homogeneity of variances was tested with Levene's test. When both assumptions were met, one‐way analysis of variance (ANOVA) was conducted, followed by Tukey's post hoc test to adjust for multiple comparisons. If the data violated the assumptions of normality or equal variances, appropriate nonparametric tests were applied instead. Statistical significance was defined as a *p* value less than 0.05. To account for potential confounding effects of covariates (e.g., body weight) on primary outcome variables (e.g., behavioral measures), ANCOVA was conducted. Following verification of the homogeneity of regression slopes, ANCOVA was performed using the car package (v.3.1‐3) and subsequently visualized with the HH package (v.3.1‐53) in R and RStudio software.

## Author Contributions

**Ying Zhang**: investigation, validation, formal analysis, visualization and writing – original draft. **Meng Teng**: investigation. **Wenxuan He**: investigation. **Lanzhou Li**: methodology, supervision. **Yongfeng Zhang**: methodology, supervision. **Shimiao Wang**: conceptualization, methodology, validation. **Chunyue Wang**: methodology, writing – review and editing, supervision, and funding acquisition. **Di Wang**: conceptualization, writing – review and editing, project administration.

## Funding

This work was supported by Natural Science Foundation of Jilin Province, China (20240101212JC).

## Conflicts of Interest

The authors declare no conflict of interest.

## Supporting information




**Supporting File**: advs74684‐sup‐0001‐SuppMat.docx.

## Data Availability

Data available on request from the authors.

## References

[1] Z. G. Zheng , S. T. Zhu , H. M. Cheng , et al., “Discovery of a Potent SCAP Degrader That Ameliorates HFD‐Induced Obesity, Hyperlipidemia and Insulin Resistance via an Autophagy‐Independent Lysosomal Pathway,” Autophagy 17, no. 7 (2021): 1592–1613, 10.1080/15548627.2020.1757955. 32432943 PMC8354609

[2] J. Ballena‐Caicedo , F. E. Zuzunaga‐Montoya , J. A. Loayza‐Castro , et al., “Global Prevalence of Dyslipidemias in the General Adult Population: A Systematic Review and Meta‐Analysis,” Journal of Health, Population and Nutrition 44, no. 1 (2025): 308, 10.1186/s41043-025-01054-3. 40859400 PMC12379389

[3] C. S. Chuang , T. Y. Yang , C. H. Muo , H. L. Su , F. C. Sung , and C. H. Kao , “Hyperlipidemia, Statin Use and the Risk of Developing Depression: A Nationwide Retrospective Cohort Study,” General Hospital Psychiatry 36, no. 5 (2014): 497–501, 10.1016/j.genhosppsych.2014.05.008. 24950917

[4] C. Chourpiliadis , Y. Zeng , A. Lovik , et al., “Metabolic Profile and Long‐Term Risk of Depression, Anxiety, and Stress‐Related Disorders,” JAMA Network Open 7, no. 4 (2024): e244525, 10.1001/jamanetworkopen.2024.4525. 38564219 PMC10988352

[5] N. Amin , J. Liu , B. Bonnechere , et al., “Interplay of Metabolome and Gut Microbiome in Individuals With Major Depressive Disorder vs Control Individuals,” JAMA Psychiatry 80, no. 6 (2023): 597–609, 10.1001/jamapsychiatry.2023.0685. 37074710 PMC10116384

[6] M. Maes , B. E. Leonard , A. M. Myint , M. Kubera , and R. Verkerk , “The New ‘5‐HT’ Hypothesis of Depression: Cell‐Mediated Immune Activation Induces Indoleamine 2,3‐Dioxygenase, Which Leads to Lower Plasma Tryptophan and an Increased Synthesis of Detrimental Tryptophan Catabolites (TRYCATs), Both of Which Contribute to the Onset of Depression,” Progress in Neuro‐Psychopharmacology & Biological Psychiatry 35, no. 3 (2011): 702–721, 10.1016/j.pnpbp.2010.12.017. 21185346

[7] K. L. Chan , F. Cathomas , and S. J. Russo , “Central and Peripheral Inflammation Link Metabolic Syndrome and Major Depressive Disorder,” Physiology 34, no. 2 (2019): 123–133, 10.1152/physiol.00047.2018. 30724127 PMC6586832

[8] Y. Kurhe , R. Mahesh , and T. Devadoss , “QCM‐4, a 5‐HT_3_ Receptor Antagonist Ameliorates Plasma HPA Axis Hyperactivity, Leptin Resistance and Brain Oxidative Stress in Depression and Anxiety‐Like Behavior in Obese Mice,” Biochemical and Biophysical Research Communications 456, no. 1 (2015): 74–79, 10.1016/j.bbrc.2014.11.036. 25446100

[9] J. Serrats , J. S. Grigoleit , E. Alvarez‐Salas , and P. E. Sawchenko , “Pro‐Inflammatory Immune‐to‐Brain Signaling Is Involved in Neuroendocrine Responses to Acute Emotional Stress,” Brain, Behavior, and Immunity 62 (2017): 53–63, 10.1016/j.bbi.2017.02.003. 28179107

[10] L. Décarie‐Spain , S. Sharma , C. Hryhorczuk , et al., “Nucleus Accumbens Inflammation Mediates Anxiodepressive Behavior and Compulsive Sucrose Seeking Elicited by Saturated Dietary Fat,” Molecular Metabolism 10 (2018): 1–13, 10.1016/j.molmet.2018.01.018. 29454579 PMC5985233

[11] H. Yadav , C. Quijano , A. K. Kamaraju , et al., “Protection From Obesity and Diabetes by Blockade of TGF‐*β*/Smad3 Signaling,” Cell Metabolism 14, no. 1 (2011): 67–79, 10.1016/j.cmet.2011.04.013. 21723505 PMC3169298

[12] S. Mitra , C. Werner , and D. M. Dietz , “Neuroadaptations and TGF‐β Signaling: Emerging Role in Models of Neuropsychiatric Disorders,” Molecular Psychiatry 27, no. 1 (2022): 296–306, 10.1038/s41380-021-01186-y. 34131268 PMC8671568

[13] A. Q. Qiu , H. Zhang , C. Q. Wang , et al., “Canonical TGF‐β Signaling Regulates the Relationship Between Prenatal Maternal Depression and Amygdala Development in Early Life,” Translational Psychiatry 11, no. 1 (2021): 170, 10.1038/s41398-021-01292-z. 33723212 PMC7961018

[14] T. T. Guo , Z. Zhang , Y. Sun , et al., “Neuroprotective Effects of Sodium Butyrate by Restoring Gut Microbiota and Inhibiting TLR4 Signaling in Mice With MPTP‐Induced Parkinson's Disease,” Nutrients 15, no. 4 (2023): 930, 10.3390/nu15040930. 36839287 PMC9960062

[15] C. Y. Liao , O. M. P. Kummert , A. M. Bair , et al., “The Autophagy Inducer Spermidine Protects Against Metabolic Dysfunction During Overnutrition,” Journals of Gerontology: Series A 76, no. 10 (2021): 1714–1725, 10.1093/gerona/glab145. PMC8436989 34060628

[16] K. Freitag , N. Sterczyk , S. Wendlinger , et al., “Spermidine Reduces Neuroinflammation and Soluble Amyloid Beta in an Alzheimer's Disease Mouse Model,” Journal of Neuroinflammation 19, no. 1 (2022): 172, 10.1186/s12974-022-02534-7. 35780157 PMC9250727

[17] B. W. Kim , S. Koppula , J. W. Kim , et al., “Modulation of LPS‐Stimulated Neuroinflammation in BV‐2 Microglia by Gastrodia Elata: 4‐Hydroxybenzyl Alcohol is the Bioactive Candidate,” Journal of Ethnopharmacology 139, no. 2 (2012): 549–557, 10.1016/j.jep.2011.11.048. 22155394

[18] K. Y. Kam , S. J. Yu , N. Jeong , et al., “ *p*‐Hydroxybenzyl Alcohol Prevents Brain Injury and Behavioral Impairment by Activating Nrf2, PDI, and Neurotrophic Factor Genes in a Rat Model of Brain Ischemia,” Molecules and Cells 31, no. 3 (2011): 209–215, 10.1007/s10059-011-0028-4. 21347705 PMC3932695

[19] S. S. Yu , J. Zhao , S. P. Lei , X. M. Lin , L. L. Wang , and Y. Zhao , “4‐Hydroxybenzyl Alcohol Ameliorates Cerebral Injury in Rats by Antioxidant Action,” Neurochemical Research 36, no. 2 (2011): 339–346, 10.1007/s11064-010-0335-8. 21120604

[20] S. Fulton , L. Décarie‐Spain , X. Fioramonti , B. Guiard , and S. Nakajima , “The Menace of Obesity to Depression and Anxiety Prevalence,” Trends in Endocrinology & Metabolism 33, no. 1 (2022): 18–35, 10.1016/j.tem.2021.10.005. 34750064

[21] W. Z. Hao , Q. Y. Ma , L. Wang , et al., “Gut Dysbiosis Induces the Development of Depression‐Like Behavior Through Abnormal Synapse Pruning in Microglia‐Mediated by Complement C3,” Microbiome 12, no. 1 (2024): 34, 10.1186/s40168-024-01756-6. 38378622 PMC10877840

[22] E. J. Lim , H. J. Kang , H. J. Jung , and E. H. Park , “Anti‐Angiogenic, Anti‐Inflammatory and Anti‐Nociceptive Activity of 4‐Hydroxybenzyl Alcohol,” Journal of Pharmacy and Pharmacology 59, no. 9 (2007): 1235–1240, 10.1211/jpp.59.9.0007. 17883894

[23] S. S. Yu , J. Zhao , W. P. Zhen , and Y. Zhao , “Neuroprotective Effect of 4‐Hydroxybenzyl Alcohol Against Transient Focal Cerebral Ischemia via Anti‐Apoptosis in Rats,” Brain Research 1308 (2010): 167–175, 10.1016/j.brainres.2009.10.037. 19857470

[24] D. Mattinzoli , M. Li , G. Castellano , et al., “Fibroblast Growth Factor 23 Level Modulates the Hepatocyte's Alpha‐2‐HS‐Glycoprotein Transcription Through the Inflammatory Pathway TNFα/NFκB,” Frontiers in Medicine 9 (2022): 1038638, 10.3389/fmed.2022.1038638. 36569120 PMC9769965

[25] S. Annett , G. Moore , and T. Robson , “FK506 Binding Proteins and Inflammation Related Signalling Pathways; Basic Biology, Current Status and Future Prospects for Pharmacological Intervention,” Pharmacology & Therapeutics 215 (2020): 107623, 10.1016/j.pharmthera.2020.107623. 32622856

[26] S. W. Yang , S. F. Chu , Q. D. Ai , et al., “Anti‐Inflammatory Effects of Higenamine (Hig) on LPS‐Activated Mouse Microglia (BV2) Through NF‐κB and Nrf2/HO‐1 Signaling Pathways,” International Immunopharmacology 85 (2020): 106629, 10.1016/j.intimp.2020.106629. 32535536

[27] Y. J. Xu , R. Zhou , J. F. Zong , et al., “Epstein‐Barr Virus‐Coded miR‐BART13 Promotes Nasopharyngeal Carcinoma Cell Growth and Metastasis via Targeting of the NKIRAS2/NF‐κB Pathway,” Cancer Letters 447 (2019): 33–40, 10.1016/j.canlet.2019.01.022. 30684592

[28] M. Maes , A. F. Almulla , Z. L. You , and Y. Q. Zhang , “Neuroimmune, Metabolic and Oxidative Stress Pathways in Major Depressive Disorder,” Nature Reviews Neurology 21, no. 9 (2025): 473–489, 10.1038/s41582-025-01116-4. 40659853

[29] H. Ahmed , Q. Leyrolle , V. Koistinen , et al., “Microbiota‐Derived Metabolites as Drivers of Gut–Brain Communication,” Gut Microbes 14, no. 1 (2022): 2102878, 10.1080/19490976.2022.2102878. 35903003 PMC9341364

[30] H. He , H. L. He , L. Mo , Z. L. You , and J. Q. Zhang , “Priming of Microglia With Dysfunctional Gut Microbiota Impairs Hippocampal Neurogenesis and Fosters Stress Vulnerability of Mice,” Brain, Behavior, and Immunity 115 (2024): 280–294, 10.1016/j.bbi.2023.10.031. 37914097

[31] H. S. Yoon , C. H. Cho , M. S. Yun , et al., “ *Akkermansia muciniphila* Secretes a Glucagon‐Like Peptide‐1‐Inducing Protein That Improves Glucose Homeostasis and Ameliorates Metabolic Disease in Mice,” Nature Microbiology 6, no. 5 (2021): 563–573, 10.1038/s41564-021-00880-5. 33820962

[32] Y. Hu , J. Zhou , and X. Q. Lin , “ *Akkermansia muciniphila* Helps in the Recovery of Lipopolysaccharide‐Fed Mice With Mild Intestinal Dysfunction,” Frontiers in Microbiology 16 (2025): 1523742, 10.3389/fmicb.2025.1523742. 40143870 PMC11938125

[33] K. F. Xu , G. Q. Wang , J. T. Gong , et al., “ *Akkermansia muciniphila* Protects Against Dopamine Neurotoxicity by Modulating Butyrate to Inhibit Microglia‐Mediated Neuroinflammation,” International Immunopharmacology 152 (2025): 114374, 10.1016/j.intimp.2025.114374. 40056512

[34] X. Ge , M. X. Zheng , M. M. Hu , et al., “Butyrate Ameliorates Quinolinic Acid‐Induced Cognitive Decline in Obesity Models,” Journal of Clinical Investigation 133, no. 4 (2023): e154612, 10.1172/jci154612. 36787221 PMC9927952

[35] S. S. Park , T. W. Kim , Y. H. Sung , Y. J. Park , M. K. Kim , and M. S. Shin , “Treadmill Exercise Ameliorates Short‐Term Memory Impairment by Suppressing Hippocampal Neuroinflammation in Poloxamer‐407‐Induced Hyperlipidemia Rats,” International Neurourology Journal 25 (2021): S81–S89, 10.5213/inj.2142342.171. 34844390 PMC8654313

[36] R. Monteiro and I. Azevedo , “Chronic Inflammation in Obesity and the Metabolic Syndrome,” Mediators of Inflammation 2010 (2010): 289645, 10.1155/2010/289645. 20706689 PMC2913796

[37] A. H. Miller and C. L. Raison , “The Role of Inflammation in Depression: From Evolutionary Imperative to Modern Treatment Target,” Nature Reviews Immunology 16, no. 1 (2016): 22–34, 10.1038/nri.2015.5. PMC5542678 26711676

[38] A. H. Miller , “Advancing an Inflammatory Subtype of Major Depression,” American Journal of Psychiatry 182, no. 6 (2025): 516–524, 10.1176/appi.ajp.20250289. 40329642 PMC12282100

[39] S. Y. Zhao , Q. Z. Wang , Z. Z. Li , et al., “LDOC1 Inhibits Proliferation and Promotes Apoptosis by Repressing NF‐κB Activation in Papillary Thyroid Carcinoma,” Journal of Experimental & Clinical Cancer Research 34 (2015): 146, 10.1186/s13046-015-0265-z. 26637328 PMC4670541

[40] E. Kiss , G. Forika , M. Dank , T. Krenacs , and Z. Nemeth , “Methyl Donors Reduce Cell Proliferation by Diminishing Erk‐Signaling and NFkB Levels, While Increasing E‐Cadherin Expression in Panc‐1 Cell Line,” International Journal of Molecular Sciences 23, no. 5 (2022): 2546, 10.3390/ijms23052546. 35269689 PMC8910410

[41] A. Henn , S. Lund , M. Hedtjärn , A. Schrattenholz , P. Pörzgen , and M. Leist , “The Suitability of BV2 Cells as Alternative Model System for Primary Microglia Cultures or for Animal Experiments Examining Brain Inflammation,” Altex‐Alternativen Zu Tierexperimenten 26, no. 2 (2009): 83–94, 10.14573/altex.2009.2.83. 19565166

[42] C. C. Lin , Y. T. Lin , S. Y. Chen , et al., “Coenzyme A Protects Against Ferroptosis via CoAlation of Mitochondrial Thioredoxin Reductase,” Journal of Clinical Investigation 135, no. 19 (2025): e190215, 10.1172/jci190215. 40694424 PMC12483566

[43] A. R. D. Delbridge , D. Huh , M. Brickelmaier , et al., “Organotypic Brain Slice Culture Microglia Exhibit Molecular Similarity to Acutely‐Isolated Adult Microglia and Provide a Platform to Study Neuroinflammation,” Frontiers in Cellular Neuroscience 14 (2020): 592005, 10.3389/fncel.2020.592005. 33473245 PMC7812919

[44] C. L. Croft , H. S. Futch , B. D. Moore , and T. E. Golde , “Organotypic Brain Slice Cultures to Model Neurodegenerative Proteinopathies,” Molecular Neurodegeneration 14, no. 1 (2019): 45, 10.1186/s13024-019-0346-0. 31791377 PMC6889333

[45] J. Hellmann‐Regen , V. Clemens , M. Grözinger , et al., “Effect of Minocycline on Depressive Symptoms in Patients With Treatment‐Resistant Depression,” JAMA Network Open 5, no. 9 (2022): e2230367, 10.1001/jamanetworkopen.2022.30367. 36103181 PMC9475381

[46] O. M. Dean , B. Kanchanatawan , M. Ashton , et al., “Adjunctive Minocycline Treatment for Major Depressive Disorder: A Proof of Concept Trial,” Australian & New Zealand Journal of Psychiatry 51, no. 8 (2017): 829–840, 10.1177/0004867417709357. 28578592

[47] M. A. Nettis , G. Lombardo , C. Hastings , et al., “Augmentation Therapy With Minocycline in Treatment‐Resistant Depression Patients With Low‐Grade Peripheral Inflammation: Results From a Double‐Blind Randomised Clinical Trial,” Neuropsychopharmacology 46, no. 5 (2021): 939–948, 10.1038/s41386-020-00948-6. 33504955 PMC8096832

[48] X. X. Zheng , C. F. Zhang , L. Q. Li , et al., “Improvement of Astrocytic Gap Junction Involves the Anti‐Depressive Effect of Celecoxib Through Inhibition of NF‐κB,” Brain Research Bulletin 207 (2024): 110871, 10.1016/j.brainresbull.2024.110871. 38211740

[49] X. Jiang , J. Hao , Z. J. Liu , et al., “Anti‐Obesity Effects of Grifola Frondosa Through the Modulation of Lipid Metabolism via Ceramide in Mice Fed a High‐Fat Diet,” Food & Function 12, no. 15 (2021): 6725–6739, 10.1039/d1fo00666e. 34160500

[50] S. S. Chen , H. H. Liu , S. M. Wang , et al., “The Neuroprotection of Verbascoside in Alzheimer's Disease Mediated Through Mitigation of Neuroinflammation via Blocking NF‐κB‐p65 Signaling,” Nutrients 14, no. 7 (2022): 1417, 10.3390/nu14071417. 35406030 PMC9003273

