# Supplementary material for: 4‐Hydroxybenzyl Alcohol Mitigates Hyperlipidemia‐Associated Depression by Inhibiting Neuroinflammation via the NKIRAS2/NF‐κB Pathway
Source: Adv Sci (Weinh). 2026 Mar 9;13(27):e17873. doi: 10.1002/advs.202517873 (PMC13170256; doi:10.1002/advs.202517873)
Supplement: Supplementary file 1 — Supporting File: advs74684‐sup‐0001‐SuppMat.docx. [file ADVS-13-e17873-s001.docx]

Supporting Information

**4-Hydroxybenzyl Alcohol Mitigates Hyperlipidemia-Associated Depression by Inhibiting Neuroinflammation via the NKIRAS2/NF-κB Pathway**

*Ying Zhang, Meng Teng, Wenxuan He, Lanzhou Li, Yongfeng Zhang, Shimiao Wang^*^, Chunyue Wang^*^, and Di Wang^*^*

**Table S1. The differential metabolites in brain between the NF group and the AHF group**

| **Metabolites** | **Mean of NF group** | **Mean of AHF group** | **VIP** | ***p* value** |
| --- | --- | --- | --- | --- |
| N-oleoyl-l-serine | 7253.893 | 11025.48407 | 1.739 | 0.034 |
| Miglitol | 161028.851 | 190072.1382 | 1.625 | 0.039 |
| Palmitoyl sphingomyelin | 414867.503 | 1050808.492 | 1.760 | 0.025 |
| Miltefosine | 10522.188 | 14426.30757 | 1.835 | 0.016 |
| Salidroside | 3049.462 | 4656.3929 | 1.744 | 0.024 |
| 1-palmitoylglycerol | 1161072.038 | 1681478.452 | 1.655 | 0.019 |
| Tolytoxin | 33136.906 | 54919.77658 | 1.793 | 0.015 |
| Hematoporphyrin | 361.546 | 1695.324197 | 1.769 | 0.027 |
| 4-hydroxybenzyl alcohol | 16702.729 | 9174.074563 | 1.735 | 0.039 |
| Malonic acid | 64193.878 | 20146.40576 | 1.704 | 0.033 |
| Valganciclovir | 68151.839 | 31846.98334 | 1.818 | 0.005 |
| Glucoraphanin | 7185.926 | 5075.160066 | 1.798 | 0.011 |
| Maleamic acid | 761048.645 | 618548.0467 | 1.682 | 0.040 |
| Maleic acid | 167471.701 | 138403.059 | 1.701 | 0.036 |
| Glyceric acid | 24078.427 | 18525.94294 | 1.769 | 0.021 |
| Vitamin c | 133510.021 | 119291.329 | 1.803 | 0.012 |
| 3-methylcytidine | 8209.741 | 4560.816227 | 1.675 | 0.018 |
| Deoxyinosine | 5881.319 | 5038.026781 | 1.684 | 0.045 |
| Oxypurinol | 36247.964 | 27967.49135 | 1.788 | 0.022 |
| Ricinoleic acid | 81967.833 | 63840.41094 | 1.686 | 0.046 |
| Tabersonine | 47837.241 | 38462.38612 | 1.866 | 0.011 |
| Inosine | 8459420.993 | 6405694.205 | 1.782 | 0.034 |
| 2-Furancarboxylic acid | 3762.356 | 2305.070548 | 1.589 | 0.048 |
| Saikosaponin a | 20440.232 | 15523.3641 | 1.744 | 0.022 |
| Mevalonic acid | 2635.675 | 1755.463727 | 1.883 | 0.007 |

Data are expressed as the mean. Differences were considered statistically significant at *p* < 0.05 and VIP > 1.

**Table S2. Differentially expressed genes in transcriptomics**

| **Number** | **symbol** | **Gene_id** | **FC (HFD vs NCD)** | **FC (HFD+4-HBA vs HFD)** |
| --- | --- | --- | --- | --- |
| Upregulated genes by 4-HBA (Number: 38) | | | | |
| 1 | *Col23a1* | ENSMUSG00000063564 | 0.282123745 | 2.021042976 |
| 2 | *Mmu-mir-3070-1* | ENSMUSG00000119229 | 0.419487579 | 2.031154525 |
| 3 | *Nox1* | ENSMUSG00000031257 | 0.422376068 | 2.145925513 |
| 4 | *Slc22a6* | ENSMUSG00000024650 | 0.499896039 | 2.229777876 |
| 5 | *Creb3l3* | ENSMUSG00000035041 | 0.440160015 | 2.235503803 |
| 6 | *Fam180a* | ENSMUSG00000047420 | 0.458883552 | 2.24326491 |
| 7 | *Slc6a13* | ENSMUSG00000030108 | 0.497648845 | 2.277893031 |
| 8 | *Gm8189* | ENSMUSG00000110298 | 0.364527611 | 2.280262635 |
| 9 | *Fmod* | ENSMUSG00000041559 | 0.41371237 | 2.314819977 |
| 10 | *Slc6a12* | ENSMUSG00000030109 | 0.430295602 | 2.483543472 |
| 11 | *Ldoc1* | ENSMUSG00000057615 | 0.428598884 | 2.53116227 |
| 12 | *Lox* | ENSMUSG00000024529 | 0.086389513 | 2.606294299 |
| 13 | *Cdh1* | ENSMUSG00000000303 | 0.447884921 | 2.706821964 |
| 14 | *Gm48710* | ENSMUSG00000111308 | 0.279418607 | 2.707572558 |
| 15 | *Dmbx1* | ENSMUSG00000028707 | 4.961925244 | 2.886457533 |
| 16 | *Casr* | ENSMUSG00000051980 | 0.273137631 | 2.978735451 |
| 17 | *Slc26a7* | ENSMUSG00000040569 | 0.455966583 | 3.085276953 |
| 18 | *Gm16796* | ENSMUSG00000085495 | 0.259247144 | 3.229028109 |
| 19 | *Plac8* | ENSMUSG00000029322 | 0.328804056 | 3.715612368 |
| 20 | *Gm44005* | ENSMUSG00000107624 | 0.315978872 | 3.82882043 |
| 21 | *Gm2446* | ENSMUSG00000112690 | 0.187764991 | 4.366871261 |
| 22 | *Gm44618* | ENSMUSG00000108905 | 0.153488237 | 4.442894858 |
| 23 | *AA414992* | ENSMUSG00000113342 | 0.089355479 | 4.551682731 |
| 24 | *Gm10591* | ENSMUSG00000096596 | 0.138696184 | 4.578896642 |
| 25 | *Gm22362* | ENSMUSG00000065036 | 0.173992859 | 5.447521555 |
| 26 | *Gm50436* | ENSMUSG00000117930 | 0.091829404 | 6.860910278 |
| 27 | *Cd28* | ENSMUSG00000026012 | 0.149011956 | 8.183965141 |
| 28 | *Gm28077* | ENSMUSG00000099715 | 0.088100865 | 10.9518296 |
| 29 | *Gm8291* | ENSMUSG00000110361 | 0.104364286 | 15.91593492 |
| 30 | *Gm44182* | ENSMUSG00000108284 | 0.061515765 | 18.18655021 |
| 31 | *Ltf* | ENSMUSG00000032496 | 0.094528947 | 18.48521605 |
| 32 | *Gm2007* | ENSMUSG00000094932 | 0.050056457 | 18.94571917 |
| 33 | *Gm28320* | ENSMUSG00000099924 | 0.036885561 | 21.20599101 |
| 34 | *Vmn1r200* | ENSMUSG00000101073 | 0.029498798 | 21.24866061 |
| 35 | *Gm3734* | ENSMUSG00000117780 | 0.037888399 | 23.467626 |
| 36 | *Gm6333* | ENSMUSG00000112423 | 0.051172056 | 25.20084144 |
| 37 | *Gm15825* | ENSMUSG00000089787 | 0.030566602 | 53.80618073 |
| 38 | *Gpr141* | ENSMUSG00000053101 | 0.037291742 | 55.7113743 |
| Downregulated genes by 4-HBA (Number: 23) | | | | |
| 1 | *Epp13* | ENSMUSG00000053367 | 15.44534295 | 0.116467554 |
| 2 | *Gm13425* | ENSMUSG00000085224 | 17.8431695 | 0.118956004 |
| 3 | *Gm42205* | ENSMUSG00000105222 | 9.372279637 | 0.138580868 |
| 4 | *Gm4631* | ENSMUSG00000078899 | 11.10317211 | 0.152925405 |
| 5 | *Bdkrb1* | ENSMUSG00000041347 | 6.793240827 | 0.231342142 |
| 6 | *Eomes* | ENSMUSG00000032446 | 11.7500908 | 0.234019394 |
| 7 | *Sln* | ENSMUSG00000042045 | 10.45339743 | 0.236973887 |
| 8 | *Gm6525* | ENSMUSG00000104043 | 2.988248238 | 0.252175496 |
| 9 | *Gm42498* | ENSMUSG00000105059 | 2.436989441 | 0.266738874 |
| 10 | *Sost* | ENSMUSG00000001494 | 13.3068759 | 0.271683716 |
| 11 | *Gm11007* | ENSMUSG00000094475 | 3.703270672 | 0.303422505 |
| 12 | *Slc38a8* | ENSMUSG00000034224 | 2.190864106 | 0.316922062 |
| 13 | *Prdm12* | ENSMUSG00000079466 | 3.310616731 | 0.330701157 |
| 14 | *Tmem95* | ENSMUSG00000094845 | 4.290661238 | 0.343075974 |
| 15 | *Smpx* | ENSMUSG00000041476 | 4.218411983 | 0.386730378 |
| 16 | *Gm30564* | ENSMUSG00000115965 | 2.265767771 | 0.395404172 |
| 17 | *Rd3* | ENSMUSG00000049353 | 2.056370185 | 0.402650492 |
| 18 | *Pik3c2g* | ENSMUSG00000030228 | 3.585563829 | 0.406943384 |
| 19 | *Igfbpl1* | ENSMUSG00000035551 | 3.234852655 | 0.413454363 |
| 20 | *Atp2a1* | ENSMUSG00000030730 | 4.661841647 | 0.42791614 |
| 21 | *Gm10076* | ENSMUSG00000060143 | 2.140132323 | 0.447698689 |
| 22 | *Kcne4* | ENSMUSG00000047330 | 2.633168784 | 0.448910579 |
| 23 | *Gm30873* | ENSMUSG00000109341 | 2.313536724 | 0.481464055 |

FC (HFD vs NCD): the ratio of gene between vehicle-treated HFD-fed mice and NCD-fed mice;

FC (HFD+4-HBA vs HFD): the ratio of gene between 4-HBA-treated HFD-fed mice and vehicle-treated HFD-fed mice.

**Table S3. Proteins with significantly different expression levels in proteomics**

| **Number** | **Protein. names** | **Unique. peptides** | **HFD/NCD Ratio** | **HFD+4-HBA/HFD Ratio** |
| --- | --- | --- | --- | --- |
| Upregulated proteins by 4-HBA (Number: 8) | | | | |
| 1 | HMG20A | 2 | 0.66369 | 1.839186 |
| 2 | NKIRAS2 | 2 | 0.659204 | 1.638706 |
| 3 | RTF2 | 2 | 0.65583 | 1.541562 |
| 4 | PUS1 | 2 | 0.644654 | 1.579794 |
| 5 | SFT2D2 | 1 | 0.62329 | 1.517444 |
| 6 | FKBPL | 2 | 0.622912 | 1.569405 |
| 7 | BET1L | 1 | 0.581867 | 1.729741 |
| 8 | SLC35F6 | 1 | 0.404638 | 2.337843 |
| Downregulated proteins by 4-HBA (Number: 3) | | | | |
| 1 | MT3 | 1 | 2.230615 | 0.602754 |
| 2 | ABHD8 | 1 | 1.660015 | 0.609904 |
| 3 | NMD3 | 2 | 1.605195 | 0.516017 |

HFD/NCD Ratio: the ratio of protein between vehicle-treated HFD-fed mice and NCD-fed mice;

HFD+4-HBA/HFD Ratio: the ratio of protein between 4-HBA-treated HFD-fed mice and vehicle-treated HFD-fed mice.**Table S4. Details of antibodies used in western blot and immunofluorescence**

| **Primary antibodies** | **Company** | **Dilution** | **Application** | **Catalog number** | **Molecular weight** |
| --- | --- | --- | --- | --- | --- |
| anti-p-IκBα(S32/36) | Affinity | 1:1000 | Western blot | AF2002 | 36 kDa |
| anti-IκBα | ABclonal | 1:1000 | Western blot | A19714 | 36 kDa |
| anti-IKKα+β | Affinity | 1:1000 | Western blot | AF6014 | 85 kDa |
| anti-p-IKKα+β(S180/181) | Affinity | 1:1000 | Western blot | AF3013 | 85 kDa |
| anti-NF-κB | Affinity | 1:1000 | Western blot | AF5006 | 65 kDa |
| anti-p-NF-κB (S536) | Affinity | 1:1000 | Western blot | AF2006 | 65 kDa |
| anti-IL-6 | Abmart | 1:1000 | Western blot | TD6087F | 24 kDa |
| anti-IL-1β | ABclonal | 1:1000 | Western blot | A22257 | 17 kDa |
| anti-TNF-α | ABclonal | 1:2000 | Western blot | A24214 | 26 kDa |
| anti-IL-4 | invtrogen | 1:1000 | Western blot | 311A9A44 | 14 kDa |
| anti-IL-10 | ABclonal | 1:800 | Western blot | A2171 | 18 kDa |
| anti-iNOS | abcam | 1:1000 | Western blot | ab15323 | 140 kDa |
| anti-NLRP3 | ABclonal | 1:1000 | Western blot | A5652 | 110 kDa |
| anti-MEK1/2 | abcam | 1:1000 | Western blot | ab131517 | 44 kDa |
| anti-p-MEK1(Ser218/222)/MEK2 (Ser222/226) | Upstate | 1:5000 | Western blot | 05-747 | 45 kDa |
| anti-p-SMAD2/3 (Thr8) | Affinity | 1:1000 | Western blot | AF3367 | 52 kDa |
| anti-SMAD2/3 | Affinity | 1:1000 | Western blot | AF6367 | 52 kDa |
| anti-TGF-β1 | ABclonal | 1:1000 | Western blot | A15103 | 44 kDa |
| anti-RAS | abcam | 1:5000 | Western blot | ab52939 | 21 kDa |
| anti-BDNF | absin | 1:1000 | Western blot | abs115532 | 40 kDa |
| anti-E-CADHERIN | Affinity | 1:1000 | Western blot | BF0219 | 135 kDa |
| anti-FKBPL | Affinity | 1:1000 | Western blot | AF0446 | 38 kDa |
| anti-NKIRAS2 | Affinity | 1:1000 | Western blot | DF2580 | 22 kDa |
| anti-LDOC1 | Affinity | 1:1000 | Western blot | DF8771 | 16 kDa |
| anti-BDKRB1 | Affinity | 1:1000 | Western blot | DF10199 | 40 kDa |
| anti-EOMES | Affinity | 1:1000 | Western blot | DF8543 | 72 kDa |
| anti-CREB3L3 | Affinity | 1:1000 | Western blot | DF2582 | 49 kDa |
| anti-CD206 | HUABIO | 1:1000 | Western blot | HA722892 | 165 kDa |
| anti-CD86 | Proteintech | 1:1000 | Western blot | 13395-1-AP | 70 kDa |
| anti-GAPDH | Affinity | 1:20000 | Western blot | AF7021 | 36 kDa |
| HRP-conjugated Goat anti-Rabbit IgG (H+L) | Tongs | 1:2000 | Western blot | NC-31460-100 | - |
| anti-PSD95 | Affinity | 1:200 | Immunofluorescence | AF7839 | 80 kDa |
| anti-SYN | Affinity | 1:200 | Immunofluorescence | AF0257 | 35 kDa |
| anti-NKIRAS2 | Affinity | 1:200 | Immunofluorescence | DF2580 | 22 kDa |
| HRP-conjugated Goat anti-Rabbit IgG (H+L) | SeraCare | 1:400 | Immunofluorescence | 5220-0336 | - |

**Table S5. Primers for RT-qPCR analysis**

| **Gene Name** | **Forward primer (5 '-3 ')** | **Reverse primer (5 '-3 ')** |
| --- | --- | --- |
| *Nkiras2* | AACCATGTCGTGGGTTCTGAG | CCGTGTATCATAGAAACGCACC |
| *iNOS* | ACATCGACCCGTCCACAGTAT | CAGAGGGGTAGGCTTGTCTC |
| *TNF-α* | TACTGAACTTCGGGGTGATTGTCC | CAGCCTTGTCCCTTGAAGAGAACC |
| *IL-1β* | TGCCACCTTTTGACAGTGATG | TGATGTGCTGCTGCGAGATT |
| *IL-6* | ACCTGTCTATACCACTTCACAAGT | AGAATTGCCATTGCACAACTCT |
| *GAPDH* | GGTTGTCTCCTGCGACTTCA | TGGTCCAGGGTTTCTTACTCC |


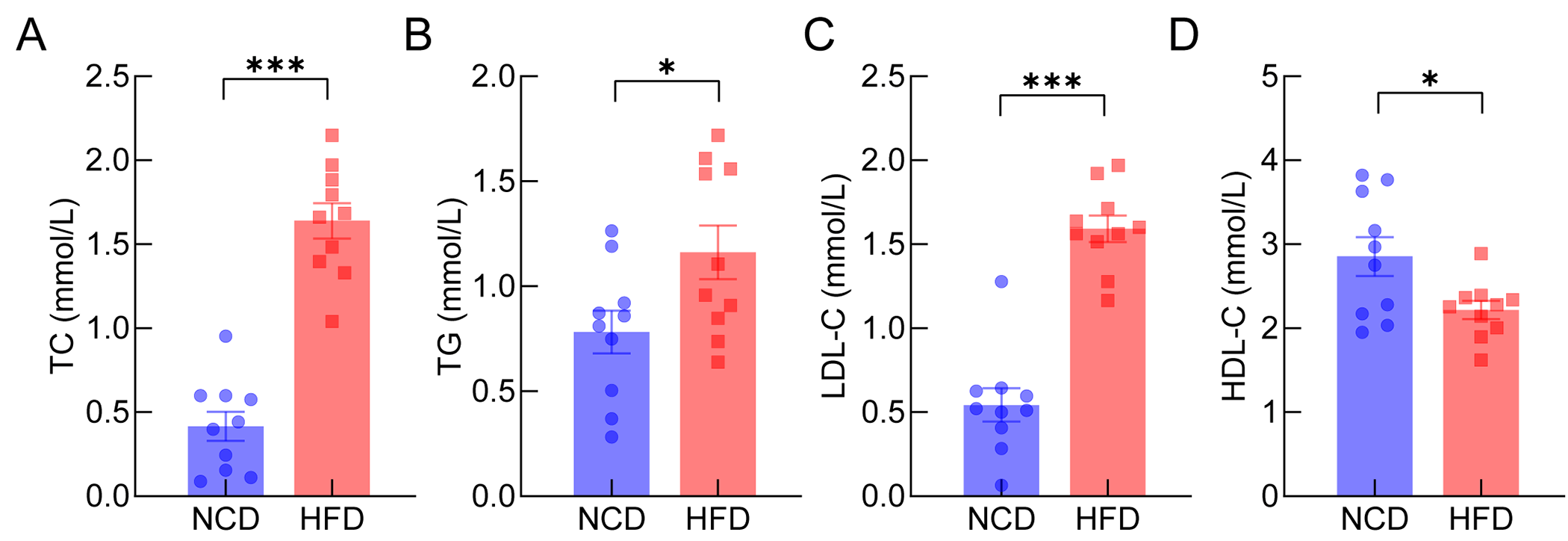


**Figure S1. Serum lipid levels in NCD-fed and HFD-fed mice.** Compared with NCD-fed mice, HFD-fed mice exhibited significantly increased serum levels of (**A**) TC (*p* < 0.0001; n = 10), (**B**) TG (*p* = 0.0322; n = 10), (**C**) LDL-C (*p* < 0.0001; n = 10), and a significantly decreased level of (**D**) HDL-C (*p* = 0.0268; n = 10). Intergroup comparisons were analyzed using a two-tailed unpaired Student’s t-test or Mann-Whitney U test to determine statistical significance. Data are expressed as mean ± SEM. **p* < 0.05 and ****p* < 0.001 vs. NCD-fed mice.


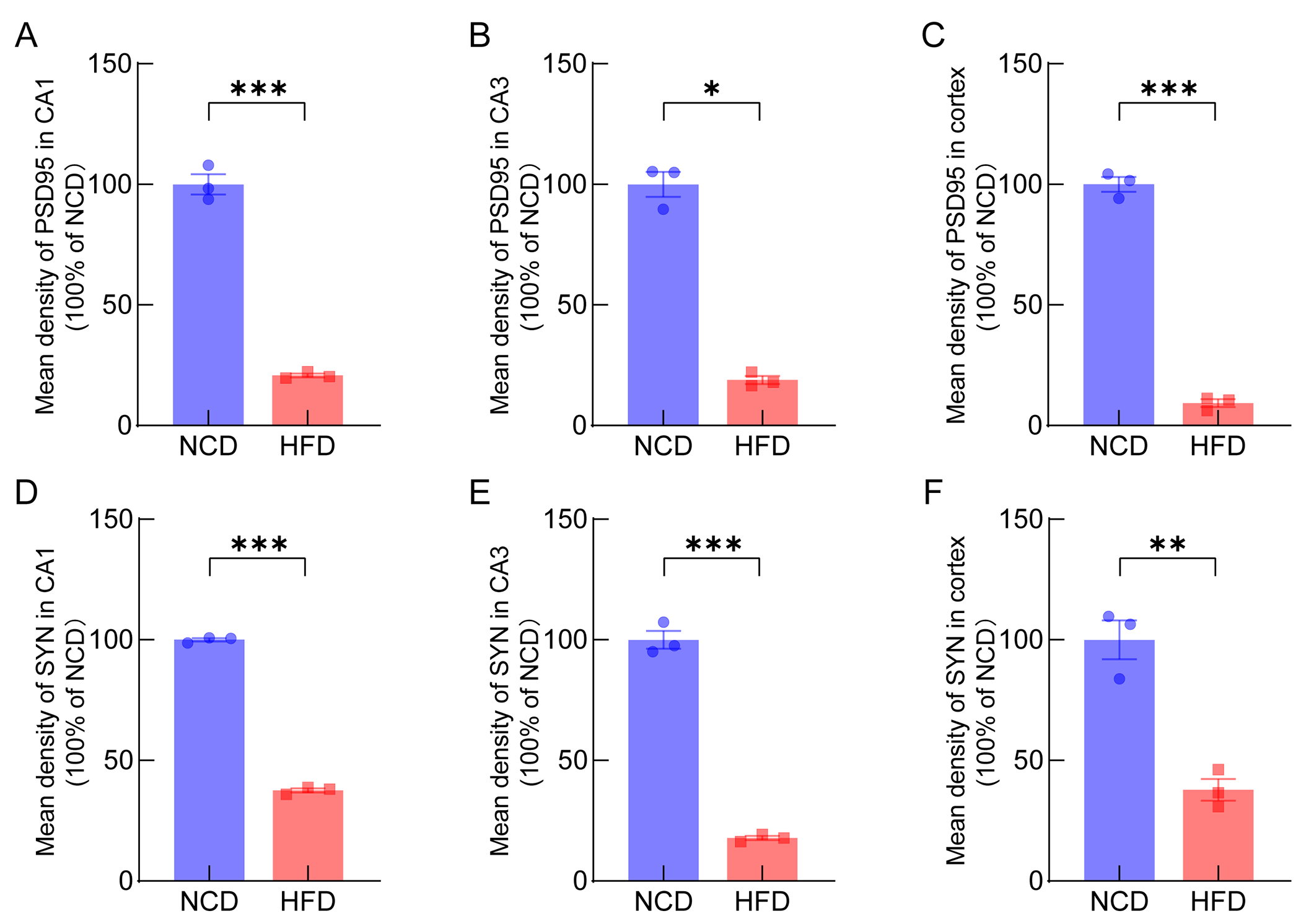


**Figure S2. The quantification of the mean fluorescence intensity of PSD95 and SYN levels.** IF semi-quantitative analysis revealed a significant reduction in PSD95 expression in (**A**) CA1 (*p* < 0.0001; n = 3), (**B**) CA3 (*p* = 0.0495; n = 3), and (**C**) cortex (*p* < 0.0001; n = 3) of HFD-fed mice. SYN expression was significantly decreased in (**D**) CA1 (*p* < 0.0001; n = 3), (**E**) CA3 (*p* < 0.0001; n = 3), and (**F**) cortex (*p* = 0.0026; n = 3) of HFD-fed mice. Intergroup comparisons were analyzed using a two-tailed unpaired Student’s t-test or Mann-Whitney U test to determine statistical significance. Data are expressed as mean ± SEM. **p* < 0.05, ***p* < 0.01, and ****p* < 0.001 *vs.* NCD-fed mice.


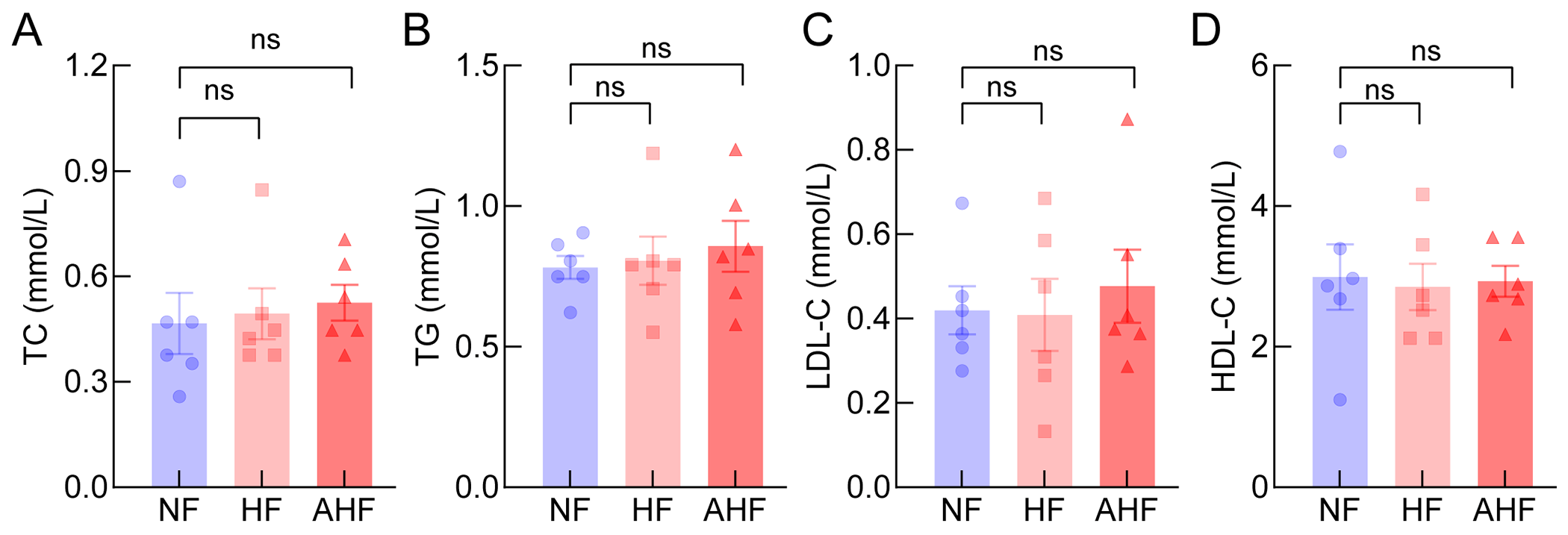


**Figure S3. Serum lipid levels in NF, HF and AHF mice.** No significant differences were observed in serum levels of (**A**) TC (HF vs. NF: *p* = 0.6279; AHF vs. NF: *p* = 0.5737; n = 6), (**B**) TG (HF vs. NF: *p* = 0.8090; AHF vs. NF: *p* = 0.4650; n = 6), (**C**) LDL-C (HF vs. NF: *p* = 0.9164; AHF vs. NF: *p* = 0.5944; n = 6), and (**D**) HDL-C (HF vs. NF: *p* = 0.8151; AHF vs. NF: *p* = 0.9133; n = 6) among NF, HF, and AHF mice. Intergroup comparisons were analyzed using a two-tailed unpaired Student’s t-test or Mann-Whitney U test to determine statistical significance. Data are expressed as mean ± SEM. ns: no statistically significant difference.


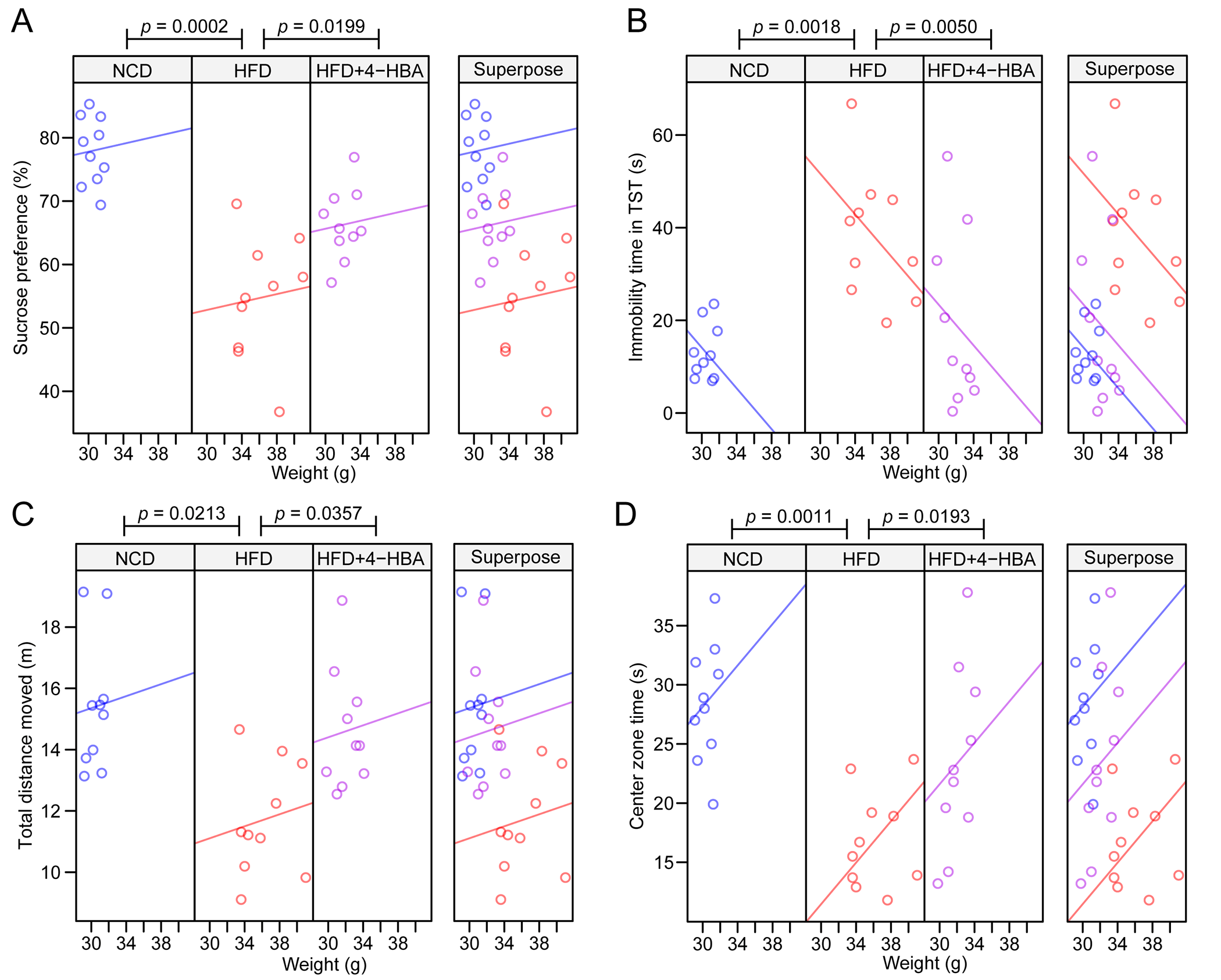


**Figure S4. Analysis of behavioral outcomes in HFD-fed mice using ANCOVA with body weight as a covariate and treatment group as a fixed factor.** (**A**) ANCOVA of sucrose preference in the SPT using body weight as a covariate and treatment group as a fixed factor (n = 10). (**B**) ANCOVA of immobility time in the TST using body weight as a covariate and treatment group as a fixed factor (n = 10). (**C**) ANCOVA of total distance moved in the OFT using body weight as a covariate and treatment group as a fixed factor (n = 10). (**D**) ANCOVA of center zone time in the OFT using body weight as a covariate and treatment group as a fixed factor (n = 10).


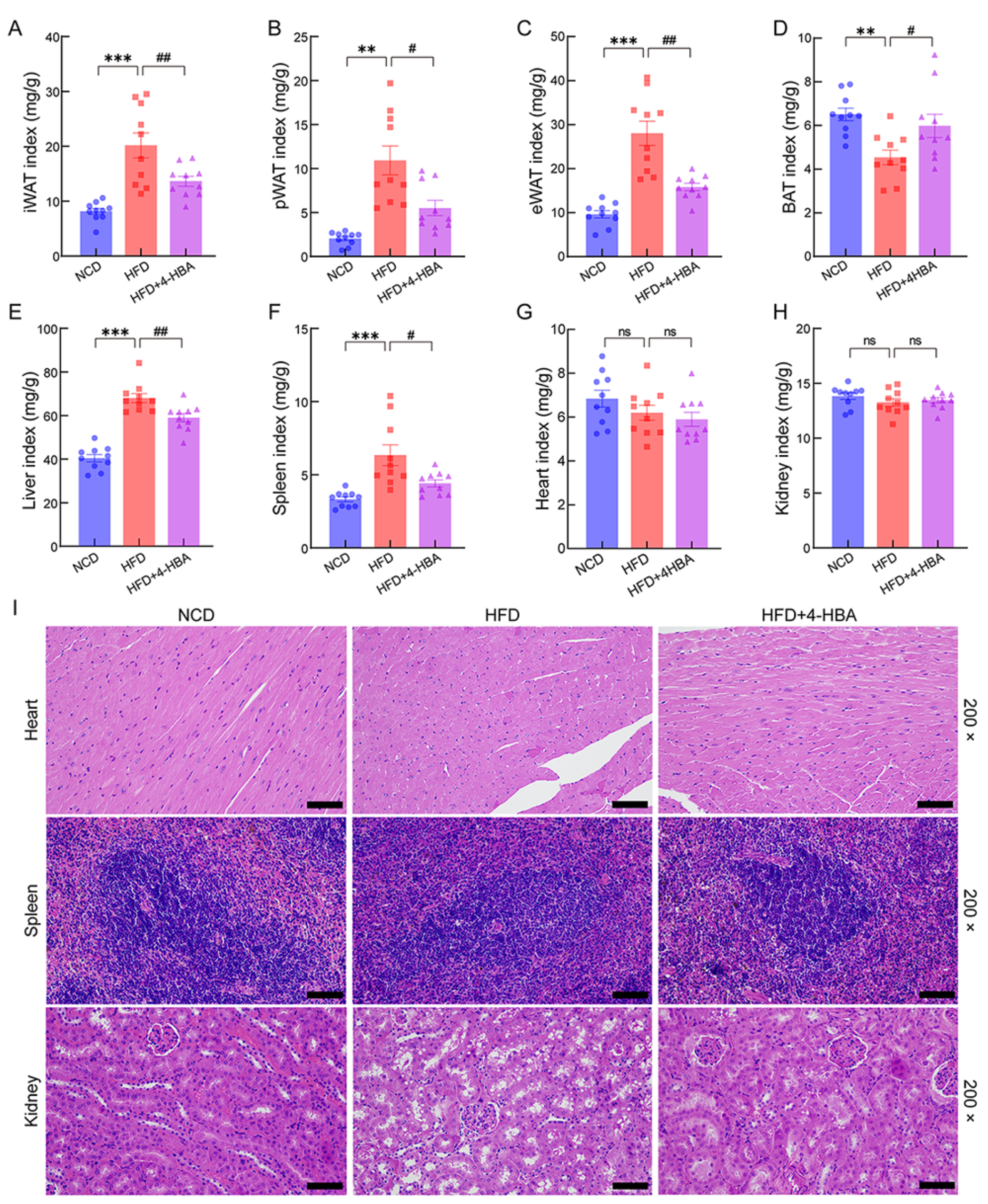


**Figure S5. *In vivo* security of 4-HBA**. 4-HBA treatment significantly decreased the (**A**) iWAT (*p* = 0.0099; n = 10), (**B**) pWAT (*p* = 0.0342; n = 10), and (**C**) eWAT (*p* = 0.0045; n = 10) indices but increased the (**D**) BAT index (*p* = 0.0423; n = 10). 4-HBA significantly reduced the (**E**) liver (*p* = 0.0066; n = 10) and (**F**) spleen (*p* = 0.0129; n = 10) indices but did not significantly affect the (**G**) heart (*p* = 0.8218; n = 10) or (**H**) kidney (*p* = 0.8753; n = 10). (**I**) H&E staining of heart, spleen, and kidney (200×, scale bar: 100 µm; n = 3). Intergroup differences were analyzed using one-way ANOVA with Tukey’s post hoc test or Tamhane’s T2 test. Data are expressed as mean ± SEM. ns: non-significant. ***p* < 0.01 and ****p* < 0.001 *vs.* NCD-fed mice; ^#^*p* < 0.05 and ^##^*p* < 0.01 *vs.* HFD-fed mice.

**
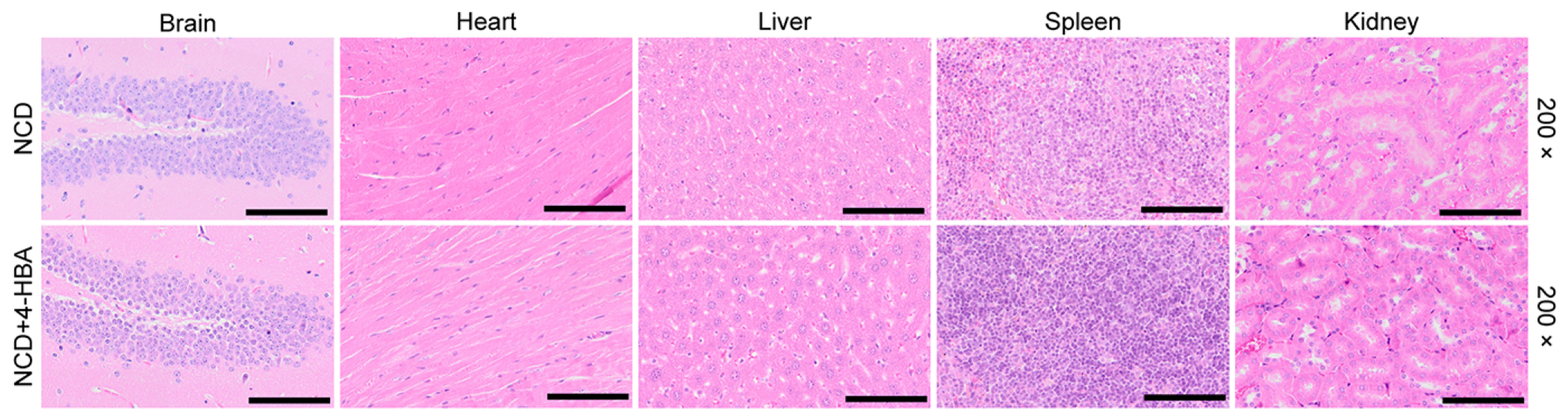
**

**Figure S6. Long-term pathological evaluation of 4-HBA.** H&E staining of brain, heart, liver, spleen, and kidney (200×, scale bar: 100 µm; n = 3).


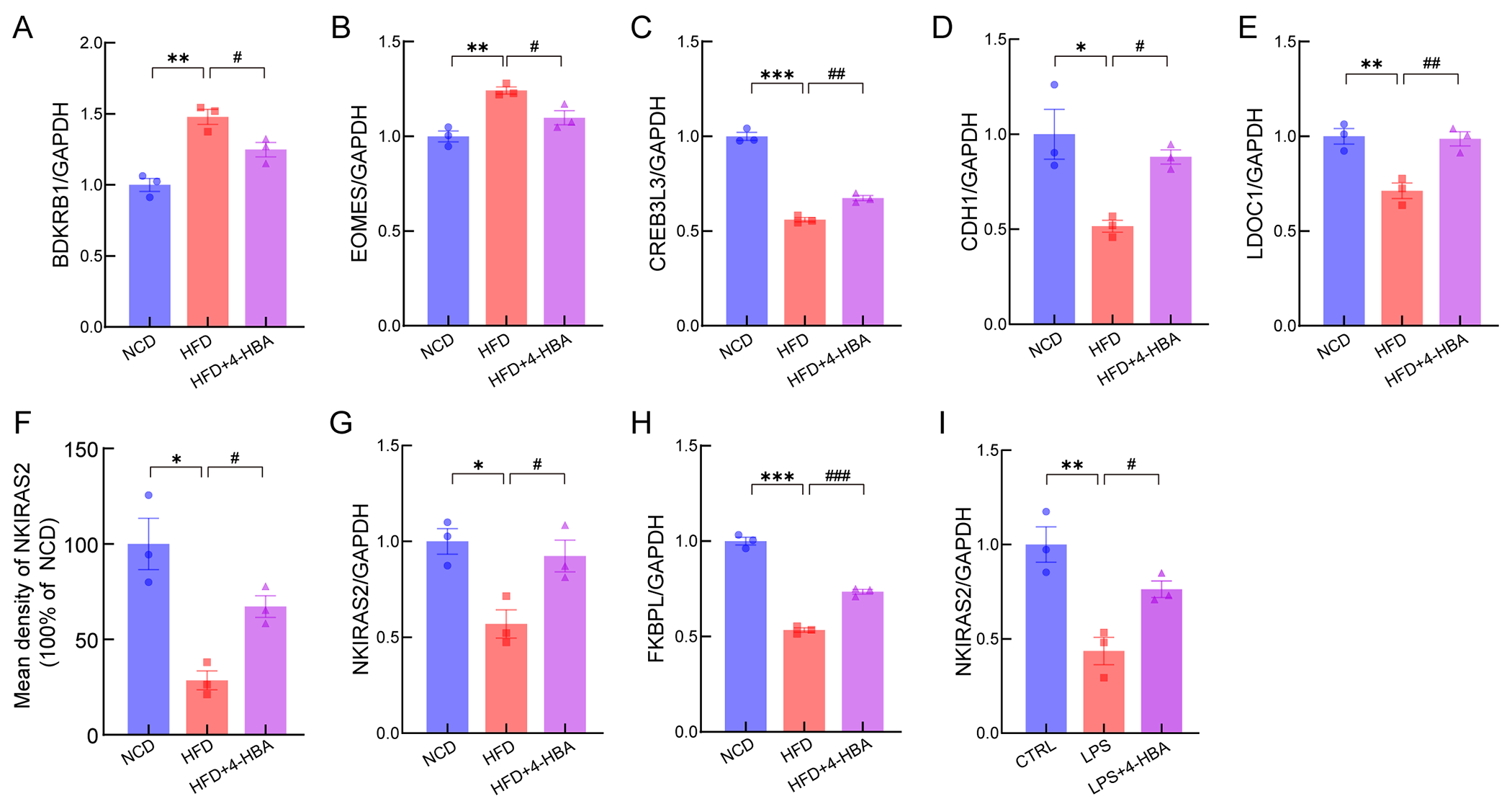


**Figure S7. Validation of differentially expressed genes and proteins.** 4-HBA treatment downregulated the protein expression of (**A**) BDKRB1 (*p* = 0.0385; n = 3) and (**B**) EOMES (*p* = 0.0303; n = 3), and upregulated (**C**) CREB3L3 (*p* = 0.0059; n = 3), (**D**) CDH1 (*p* = 0.0437; n = 3), and (**E**) LDOC1 (*p* = 0.0068; n = 3) in the brains of HFD-fed mice. (**F**) 4-HBA treatment significantly upregulated NKIRAS2 expression in the brains of HFD-fed mice (*p* = 0.0154; n = 3). 4-HBA treatment upregulated the protein expression of (**G**) NKIRAS2 (*p* = 0.0353; n = 3) and (**H**) FKBPL (*p* = 0.0002; n = 3) in the brains of HFD-fed mice. (**I**) 4-HBA treatment significantly upregulated NKIRAS2 protein expression in LPS-stimulated BV2 cells (*p* = 0.0442; n = 3). Intergroup differences were analyzed using one-way ANOVA with Tukey’s post hoc test or Tamhane’s T2 test. Data are expressed as mean ± SEM. **p* < 0.05, ***p* < 0.01, and ****p* < 0.001 *vs.* NCD-fed mice; ^#^*p* < 0.05, ^##^*p* < 0.01, and ^###^*p* < 0.001 *vs.* HFD-fed mice for **A-H**. ***p* < 0.01 *vs.* CTRL BV2 cells; ^#^*p* < 0.05 *vs.* LPS-stimulated BV2 cells for **I**.


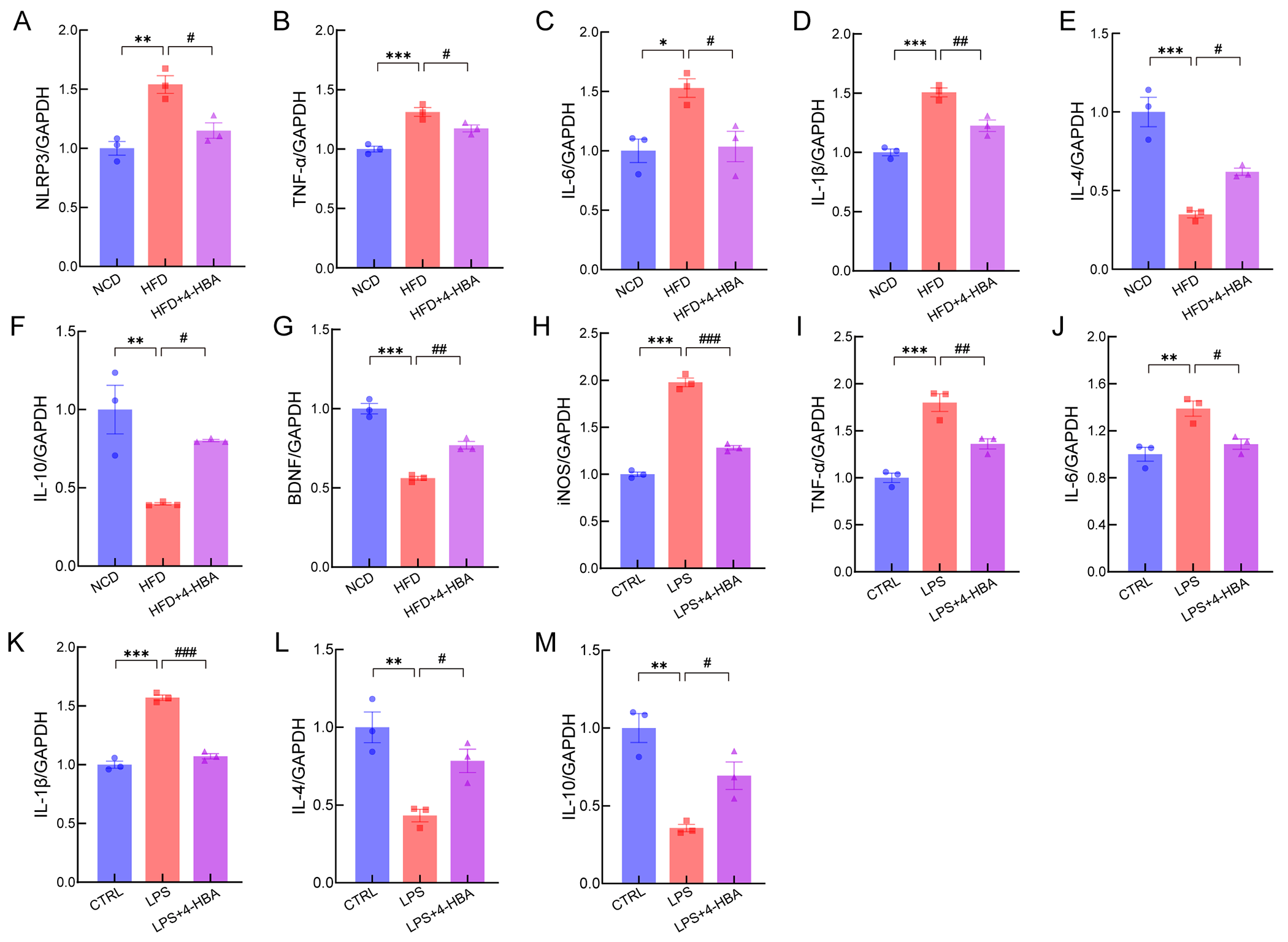


**Figure S8. Validation of inflammatory factors.** 4-HBA treatment downregulated the protein expression of (**A**) NLRP3 (*p* = 0.0141; n = 3), (**B**) TNF-α (*p* = 0.0412; n = 3), (**C**) IL-6 (*p* = 0.0347; n = 3), (**D**) IL-1β (*p* = 0.0054; n = 3), and upregulated (**E**) IL-4 (*p* = 0.0351; n = 3), (**F**) IL-10 (*p* = 0.0440; n = 3), (**G**) BDNF (*p* = 0.0022; n = 3) in the brains of HFD-fed mice. 4-HBA treatment downregulated the protein expression of (**H**) iNOS (*p* < 0.0001; n = 3), (**I**) TNF-α (*p* = 0.0096; n = 3), (**J**) IL-6 (*p* = 0.0218; n = 3), (**K**) IL-1β (*p* < 0.0001; n = 3), and upregulated (**L**) IL-4 (*p* = 0.0375; n = 3), (**M**) IL-10 (*p* = 0.0444; n = 3) in LPS-stimulated BV2 cells. Intergroup differences were analyzed using one-way ANOVA with Tukey’s post hoc test or Tamhane’s T2 test. Data are expressed as mean ± SEM. **p* < 0.05, ***p* < 0.01, and ****p* < 0.001 *vs.* NCD-fed mice; ^#^*p* < 0.05 and ^##^*p* < 0.01 *vs.* HFD-fed mice for **A-G**. ***p* < 0.01 and ****p* < 0.001 *vs.* CTRL BV2 cells; ^#^*p* < 0.05, ^##^*p* < 0.01, and ^###^*p* < 0.001 *vs.* LPS-stimulated BV2 cells for **H-M**.


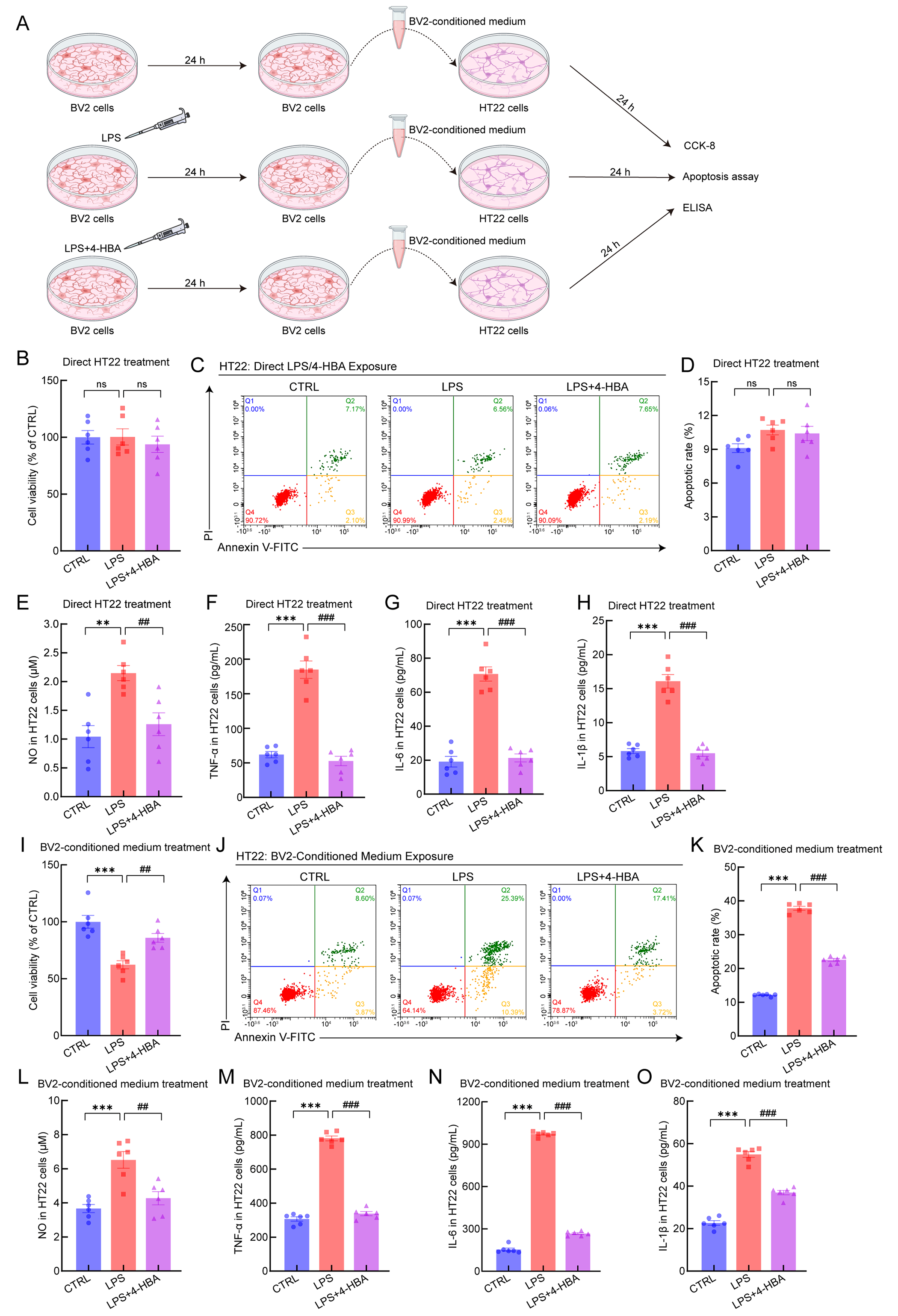


**Figure S9. Effects of direct LPS/4-HBA stimulation and BV2-conditioned medium on HT22 neuronal cells**. (**A**) Schematic illustration of the BV2-conditioned medium stimulation system. (**B**) Direct exposure of HT22 cells to LPS (*p* = 0.9992; n = 6) or 4-HBA (*p* = 0.7753; n = 6) did not significantly alter cell viability. (**C, D**) Apoptosis in HT22 cells was not significantly altered by LPS (*p* = 0.0871; n = 6) or 4-HBA (*p* = 0.8982; n = 6). 4-HBA treatment significantly reduced (**E**) NO (*p* = 0.0076; n = 6), (**F**) TNF-α (*p* < 0.0001; n = 6), (**G**) IL-6 (*p* < 0.0001; n = 6), and (**H**) IL-1β (*p* = 0.0001; n = 6) in LPS-stimulated HT22 supernatants. (**I**) Conditioned medium from BV2 cells co-treated with LPS and 4-HBA restored HT22 cell viability (*p* = 0.0049; n = 6). (**J, K**) Conditioned medium from BV2 cells co-treated with LPS and 4-HBA significantly attenuated apoptosis (*p* < 0.0001; n = 6). Conditioned medium from BV2 cells co-treated with LPS and 4-HBA markedly decreased (**L**) NO (*p* = 0.0025; n = 6), (**M**) TNF-α (*p* < 0.0001; n = 6), (**N**) IL-6 (*p* < 0.0001; n = 6), and (**O**) IL-1β (*p* < 0.0001; n = 6) levels in HT22 supernatants. Intergroup differences were analyzed using one-way ANOVA with Tukey’s post hoc test or Tamhane’s T2 test. Data are expressed as mean ± SEM. ns: no statistically significant difference. ***p* < 0.01 and ****p* < 0.001 *vs.* CTRL HT22 cells; ^##^*p* < 0.01 and ^###^*p* < 0.001 *vs.* LPS-stimulated HT22 cells for **B-H**. ****p* < 0.001 *vs.* HT22 cells treated with conditioned medium from untreated BV2 cells; ^##^*p* < 0.01 and ^###^*p* < 0.001 *vs.* HT22 cells treated with conditioned medium from LPS-stimulated BV2 cells for **I-O.**


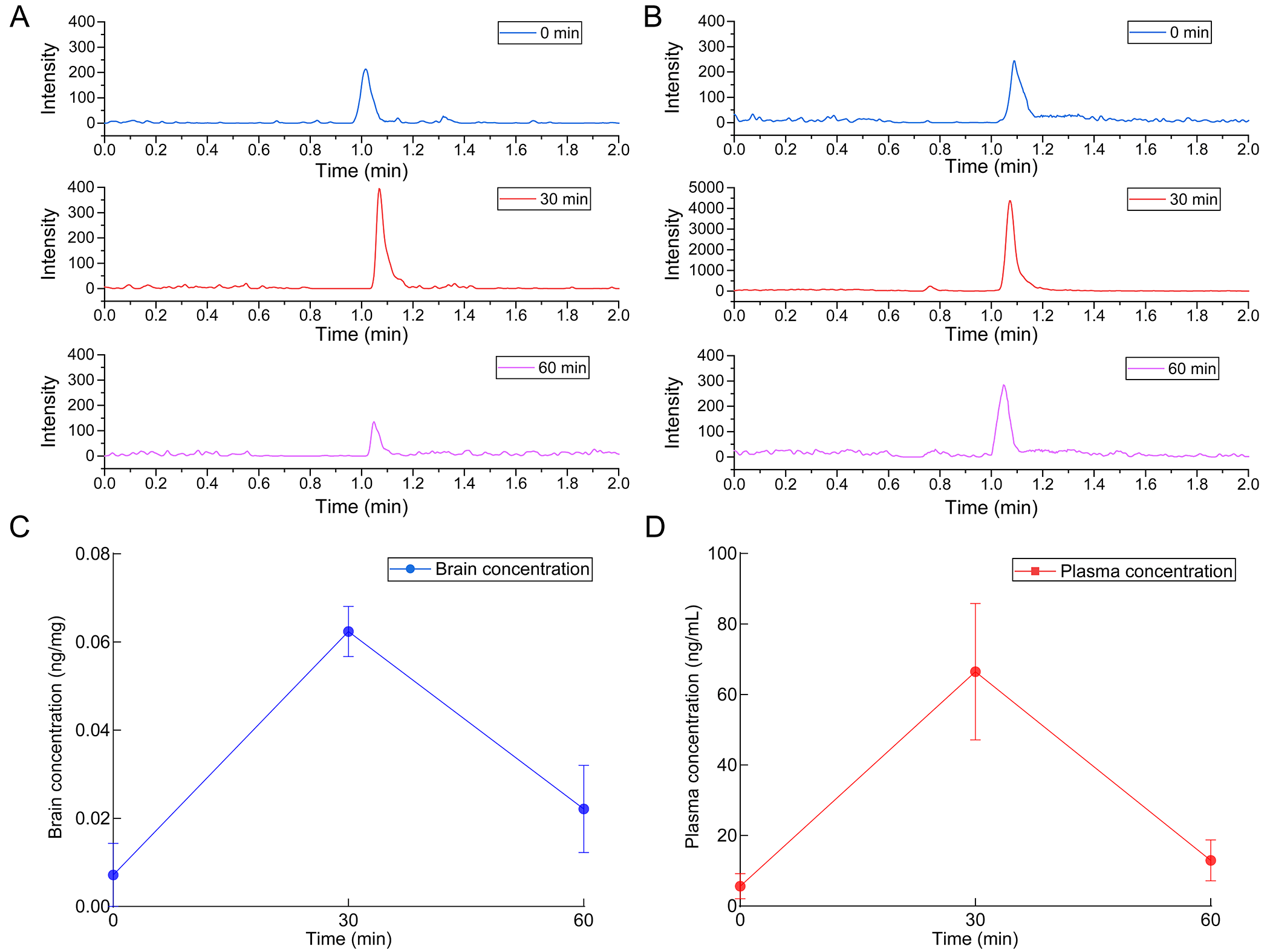


**Figure S10. Evidence for blood-brain barrier penetration of 4-HBA.** (**A**) Representative chromatograms of 4-HBA detected in brain tissue (n = 6). (**B**) Representative chromatograms of 4-HBA detected in plasma (n = 6). (**C**) Quantitative analysis of 4-HBA concentrations in brain tissue (n = 6). (**D**) Quantitative analysis of 4-HBA concentrations in plasma (n = 6).


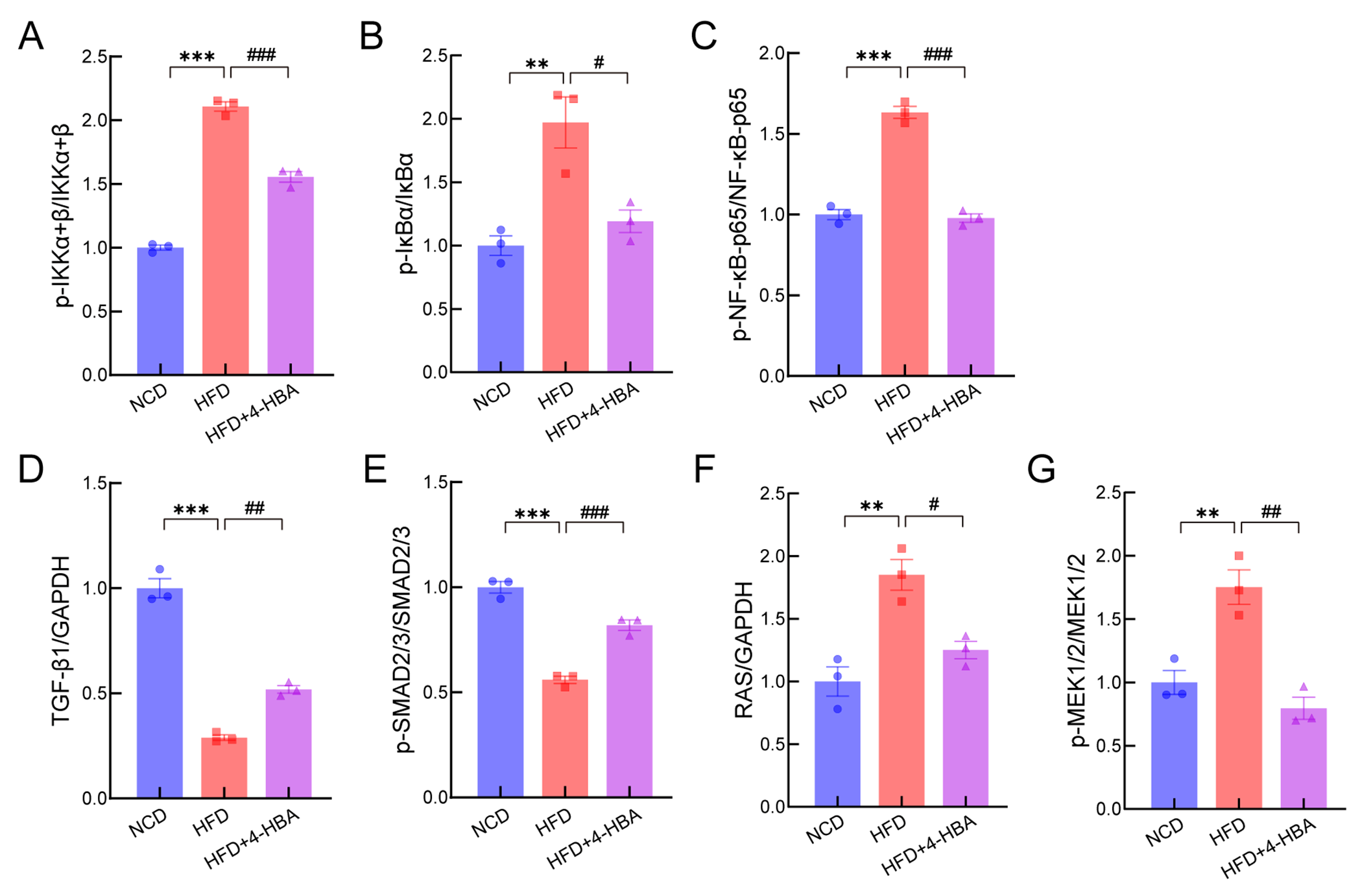


**Figure S11. 4-HBA regulated the NF-κB, TGF-β1/SMAD2/3, and RAS/MEK pathways in HFD-fed mice.** In brain tissue, 4-HBA treatment reduced (**A**) p-IKKα+β (*p* = 0.0001; n = 3), (**B**) p-IκBα (*p* = 0.0150; n = 3), and (**C**) p-NF-κB-p65 (*p* < 0.0001; n = 3); increased (**D**) TGF-β1 (*p* = 0.0036; n = 3) and (**E**) p-SMAD2/3 (*p* = 0.0006; n = 3); decreased (**F**) RAS (*p* = 0.0165; n = 3) and (**G**) p-MEK1/2 (*p* = 0.0018; n = 3). Intergroup differences were analyzed using one-way ANOVA with Tukey’s post hoc test or Tamhane’s T2 test. Data are expressed as mean ± SEM. ***p* < 0.01 and ****p* < 0.001 *vs.* NCD-fed mice; ^#^*p* < 0.05, ^##^*p* < 0.01, and ^###^*p* < 0.001 *vs.* HFD-fed mice.


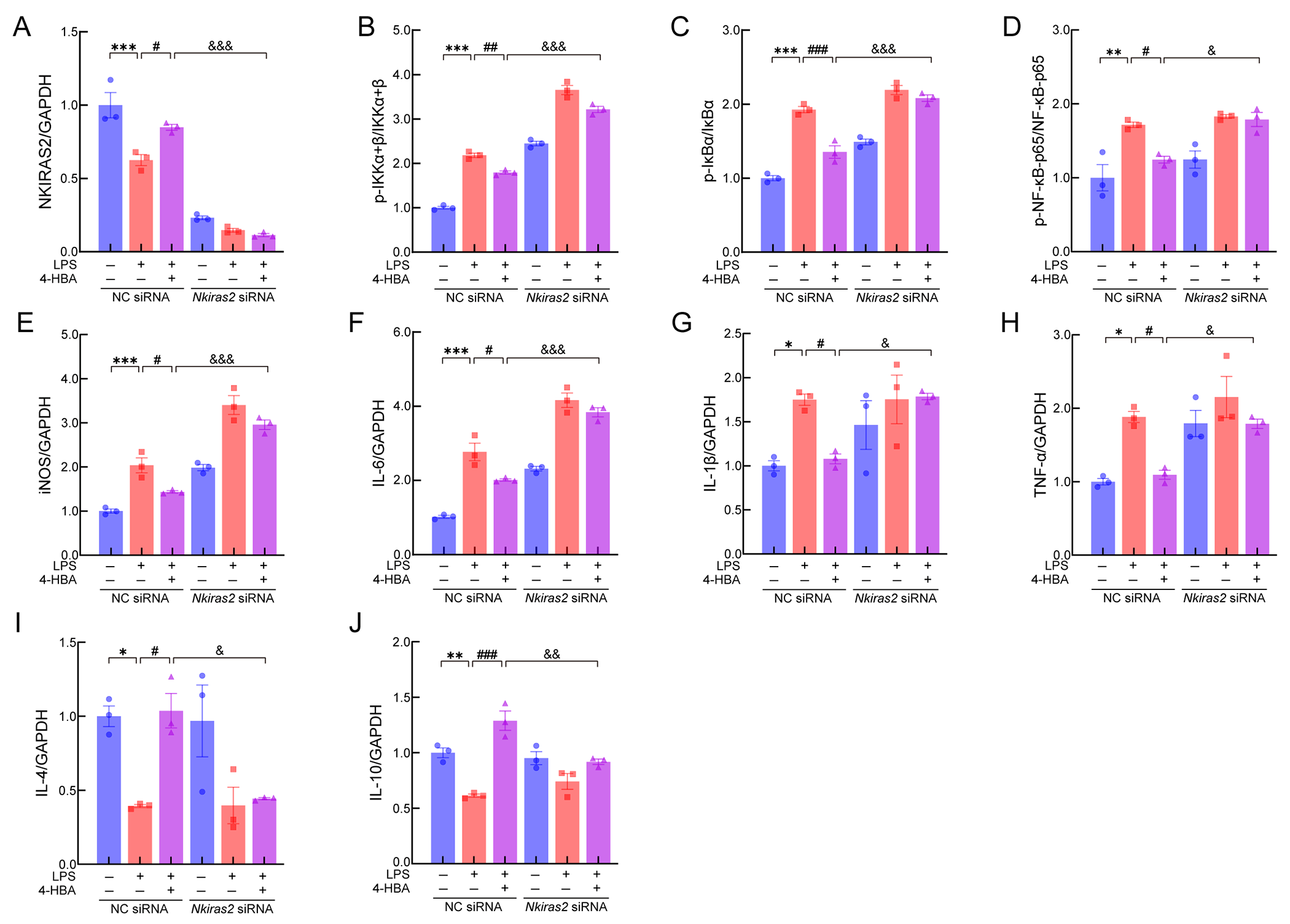


**Figure S12. 4-HBA attenuated neuroinflammatory responses via the NKIRAS2/NF-κB pathway in BV2 cells.** *Nkiras2* silencing abolished the effects of 4-HBA on (**A**) NKIRAS2 (*p* < 0.0001; n = 3), (**B**) p-IKKα+β (*p* < 0.0001; n = 3), (**C**) p-IκBα (*p* < 0.0001; n = 3), (**D**) p-NF-κB-p65 (*p* = 0.0203; n = 3), (**E**) iNOS (*p* < 0.0001; n = 3), (**F**) IL-6 (*p* < 0.0001; n = 3), (**G**) IL-1β (*p* = 0.0137; n = 3), (**H**) TNF-α (*p* = 0.0217; n = 3), (**I**) IL-4 (*p* = 0.0483; n = 3), and (**J**) IL-10 (*p* = 0.0055; n = 3) compared with NC siRNA treated cells. Intergroup differences were analyzed using one-way ANOVA with Tukey’s post hoc test or Tamhane’s T2 test. Data are expressed as mean ± SEM. **p* < 0.05, ***p* < 0.01, and ****p* < 0.001 *vs.* CTRL BV2 cells transfected with NC siRNA; ^#^*p* < 0.05, ^##^*p* < 0.01, and ^###^*p* < 0.001 *vs.* LPS-stimulated BV2 cells transfected with NC siRNA; ^&^*p* < 0.05, ^&&^*p* < 0.01, and ^&&&^*p* < 0.001 *vs.* 4-HBA-treated BV2 cells transfected with NC siRNA.


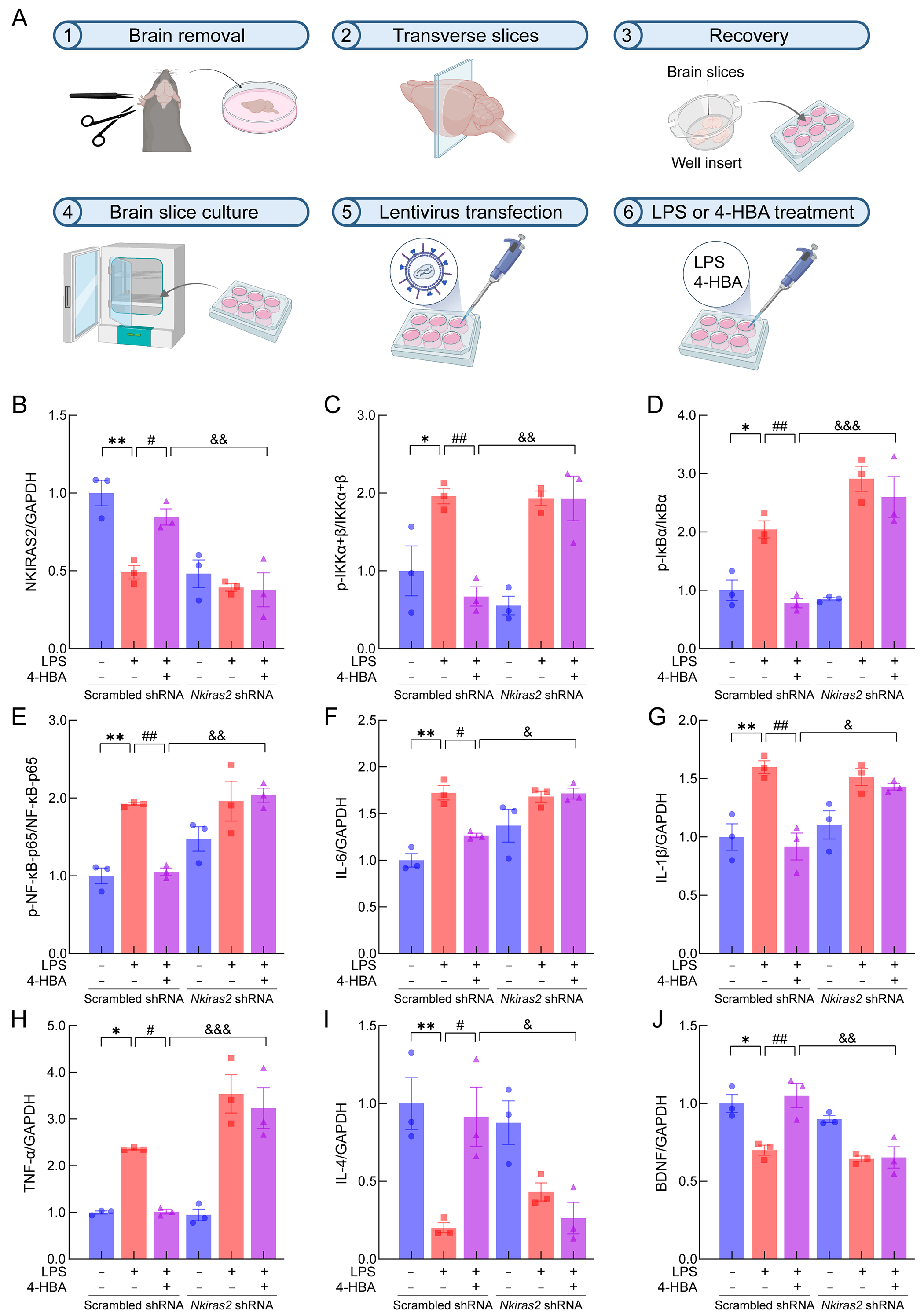


**Figure S13. 4-HBA attenuated neuroinflammatory responses via the NKIRAS2/NF-κB pathway in OBSCs.** (**A**) Schematic illustration of the experimental workflow for OBSCs. *Nkiras2* silencing abolished the effects of 4-HBA on (**B**) NKIRAS2 (*p* = 0.0066; n = 3), (**C**) p-IKKα+β (*p* = 0.0070; n = 3), (**D**) p-IκBα (*p* = 0.0003; n = 3), (**E**) p-NF-κB-p65 (*p* = 0.0029; n = 3), (**F**) IL-6 (*p* = 0.0406; n = 3), (**G**) IL-1β (*p* = 0.0183; n = 3), (**H**) TNF-α (*p* = 0.0005; n = 3), (**I**) IL-4 (*p* = 0.0330; n = 3), and (**J**) BDNF (*p* = 0.0016; n = 3) compared with scrambled shRNA treated OBSCs. Intergroup differences were analyzed using one-way ANOVA with Tukey’s post hoc test or Tamhane’s T2 test. Data are expressed as mean ± SEM. **p* < 0.05 and ***p* < 0.01 *vs.* CTRL OBSCs transfected with scrambled shRNA; ^#^*p* < 0.05 and ^##^*p* < 0.01 *vs.* LPS-stimulated OBSCs transfected with scrambled shRNA; ^&^*p* < 0.05, ^&&^*p* < 0.01, and ^&&&^*p* < 0.001 *vs.* 4-HBA treated OBSCs transfected with scrambled shRNA.


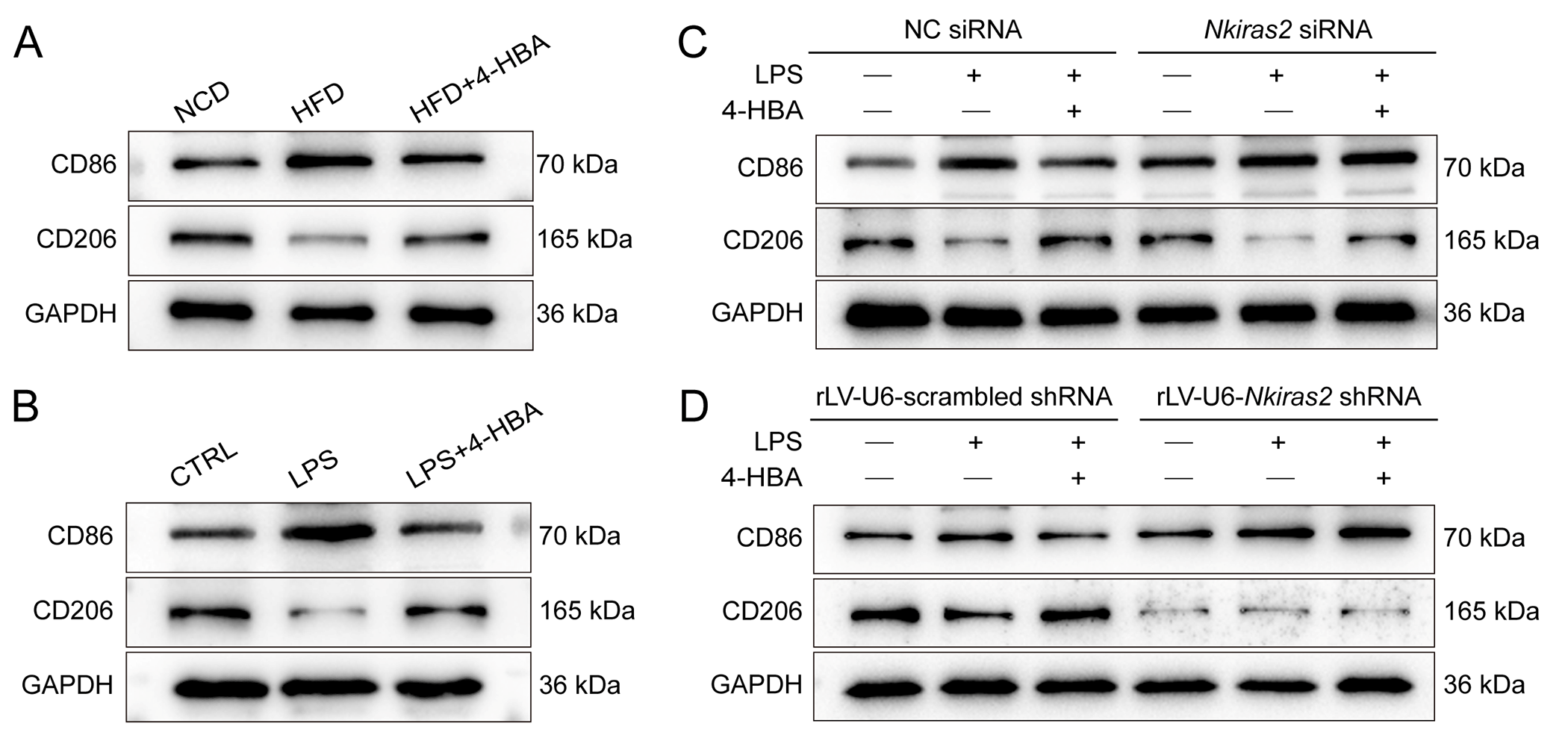


**Figure S14. 4-HBA modulated microglial M1/M2 polarization via the NKIRAS2/NF-κB pathway.** (**A**) Representative Western blot images showed CD86 and CD206 protein levels in brain tissues from HFD-fed mice treated with 4-HBA (n = 3). (**B**) Representative Western blot images showed CD86 and CD206 protein levels in LPS-stimulated BV2 cells (n = 3). (**C**) Representative Western blot images showed CD86 and CD206 protein levels in BV2 cells transfected with *Nkiras2* siRNA or NC siRNA (n = 3). (**D**) Representative Western blot images showed CD86 and CD206 protein levels in OBSCs transfected with *Nkiras2* shRNA or scrambled siRNA (n = 3).


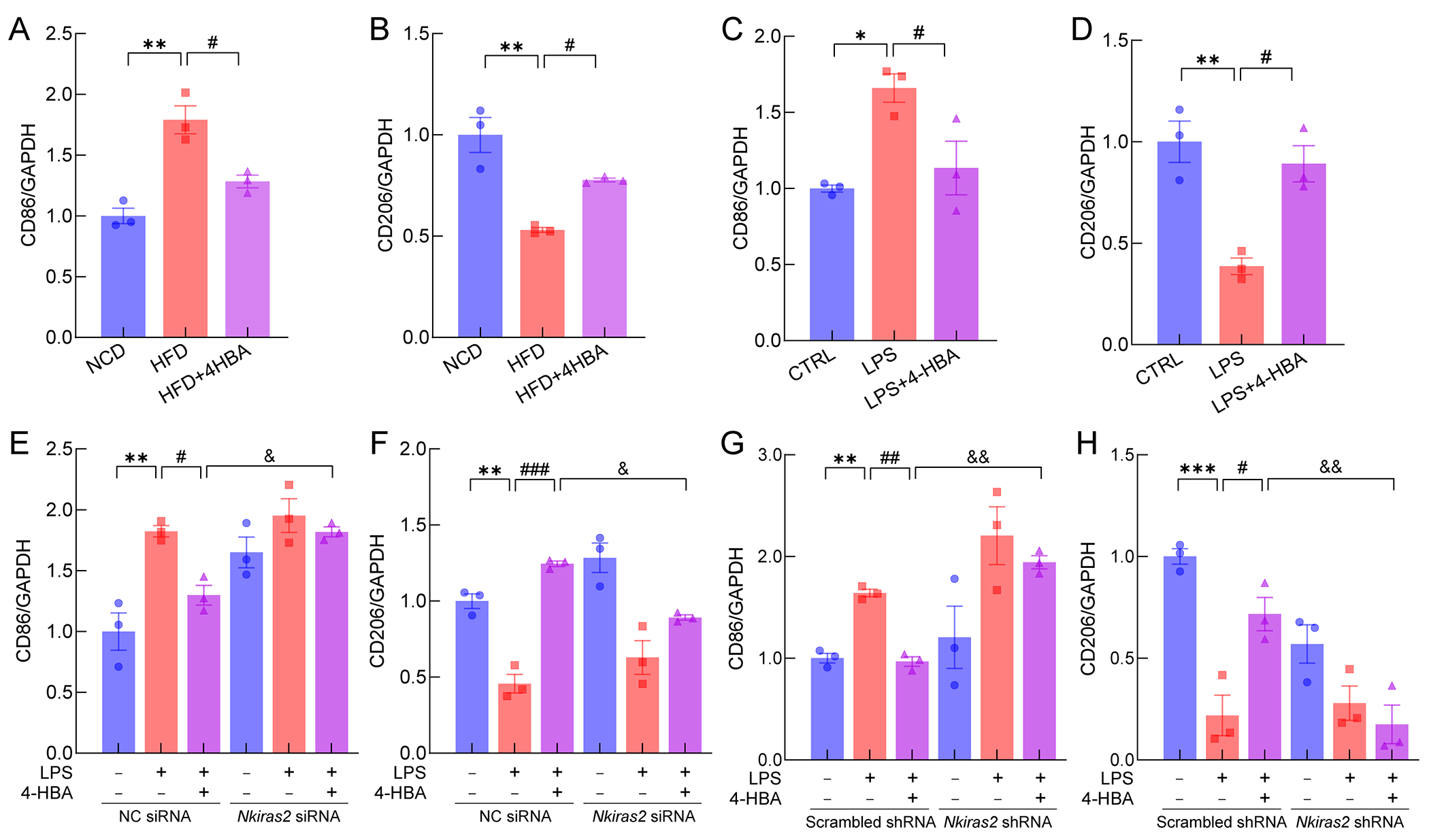


**Figure S15. Western blot quantification of 4-HBA regulated microglial M1/M2 polarization.** 4-HBA decreased (**A**) CD86 (*p* = 0.0110; n = 3) and increased (**B**) CD206 (*p* = 0.0320; n = 3) in the brains of HFD-fed mice. 4-HBA decreased (**C**) CD86 (*p* = 0.0424; n = 3) and increased (**D**) CD206 (*p* = 0.0111; n = 3) in LPS-stimulated BV2 cells. *Nkiras2* silencing abolished the effects of 4-HBA on (**E**) CD86 (*p* = 0.0436; n = 3) and (**F**) CD206 (*p* = 0.0309; n = 3) in BV2 cells. *Nkiras2* silencing abolished the effects of 4-HBA on (**G**) CD86 (*p* = 0.0064; n = 3) and (**H**) CD206 (*p* = 0.0071; n = 3) in OBSCs. Intergroup differences were analyzed using one-way ANOVA with Tukey’s post hoc test or Tamhane’s T2 test. Data are expressed as mean ± SEM. ***p* < 0.01 *vs.* NCD-fed mice; ^#^*p* < 0.05 *vs.* HFD-fed mice for **A**-**B**. **p* < 0.05 and ***p* < 0.01 *vs.* CTRL BV2 cells; ^#^*p* < 0.05 *vs.* LPS-stimulated BV2 cells for **C-D**. ***p* < 0.01 *vs.* CTRL BV2 cells transfected with NC siRNA; ^#^*p* < 0.05 and ^###^*p* < 0.001 *vs.* LPS-stimulated BV2 cells transfected with NC siRNA; ^&&&^*p* < 0.001 *vs.* 4-HBA-treated BV2 cells transfected with NC siRNA for **E-F**. ***p* < 0.01 and ****p* < 0.001 *vs.* CTRL OBSCs transfected with scrambled shRNA; ^#^*p* < 0.05 and ^##^*p* < 0.01 *vs.* LPS-stimulated OBSCs transfected with scrambled shRNA; ^&&^*p* < 0.01 *vs.* 4-HBA treated OBSCs transfected with scrambled shRNA for **G-H**.


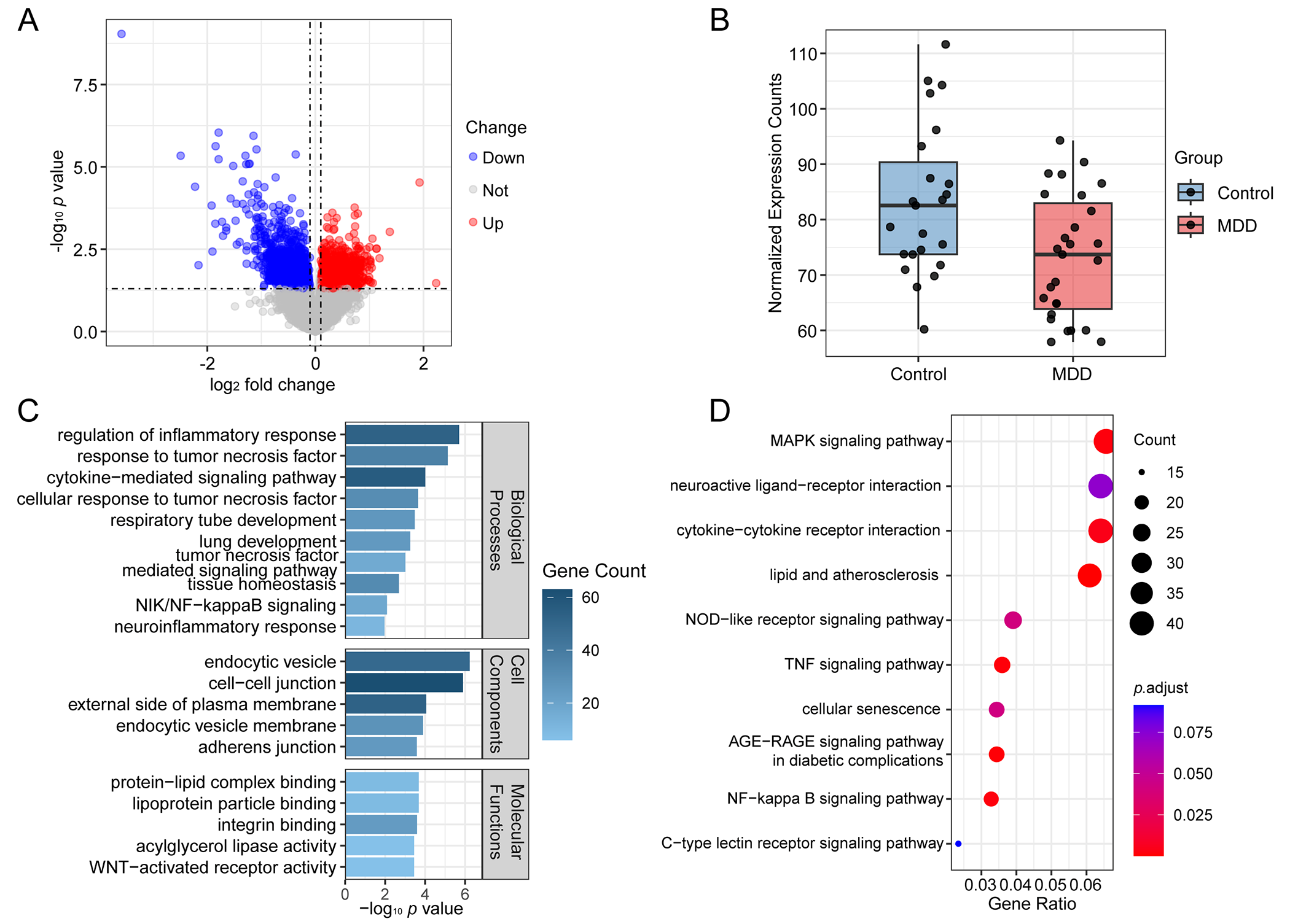


**Figure S16. Transcriptomic analysis of human MDD datasets.** (**A**) Volcano plot illustrating DEGs between MDD patients and healthy controls. (**B**) Comparative expression levels of *NKIRAS2* in healthy controls and MDD patients (*p* = 0.0166). (**C**) GO enrichment analysis of DEGs. (**D**) KEGG pathway enrichment analysis of DEGs.
